# The Arginine Pairs and C-Termini of the Sso7c4 from *Sulfolobus solfataricus* Participate in Binding and Bending DNA

**DOI:** 10.1371/journal.pone.0169627

**Published:** 2017-01-09

**Authors:** Bo-Lin Lin, Chin-Yu Chen, Chun-Hsiang Huang, Tzu-Ping Ko, Cheng-Hung Chiang, Kuan-Fu Lin, Yuan-Chih Chang, Po-Yen Lin, Hui-Hsu Gavin Tsai, Andrew H.-J. Wang

**Affiliations:** 1 Institute of Biological Chemistry, Academia Sinica, Taipei, Taiwan; 2 Institute of Bioinformatics and Structural Biology, National Tsing-Hua University, Hsinchu, Taiwan; 3 Department of Life Sciences, National Central University, Taoyuan, Taiwan; 4 Protein Diffraction Group, Experimental Facility Division, National Synchrotron Radiation Research Center, Hsinchu, Taiwan; 5 Institute of Cellular and Organismic Biology, Academia Sinica, Taipei, Taiwan; 6 Department of Chemistry, National Central University, Taoyuan, Taiwan; University of Manchester, UNITED KINGDOM

## Abstract

The Sso7c4 from *Sulfolobus solfataricus* forms a dimer, which is believed to function as a chromosomal protein involved in genomic DNA compaction and gene regulation. Here, we present the crystal structure of wild-type Sso7c4 at a high resolution of 1.63 Å, showing that the two basic C-termini are disordered. Based on the fluorescence polarization (FP) binding assay, two arginine pairs, R11/R22′ and R11′/R22, on the top surface participate in binding DNA. As shown in electron microscopy (EM) images, wild-type Sso7c4 compacts DNA through bridging and bending interactions, whereas the binding of C-terminally truncated proteins rigidifies and opens DNA molecules, and no compaction of the DNA occurs. Moreover, the FP, EM and fluorescence resonance energy transfer (FRET) data indicated that the two basic and flexible C-terminal arms of the Sso7c4 dimer play a crucial role in binding and bending DNA. Sso7c4 has been classified as a repressor-like protein because of its similarity to *Escherichia coli* Ecrep 6.8 and Ecrep 7.3 as well as *Agrobacterium tumefaciens* ACCR in amino acid sequence. Based on these data, we proposed a model of the Sso7c4-DNA complex using a curved DNA molecule in the catabolite activator protein-DNA complex. The DNA end-to-end distance measured with FRET upon wild-type Sso7c4 binding is almost equal to the distance measured in the model, which supports the fidelity of the proposed model. The FRET data also confirm the EM observation showing that the binding of wild-type Sso7c4 reduces the DNA length while the C-terminal truncation does not. A functional role for Sso7c4 in the organization of chromosomal DNA and/or the regulation of gene expression through bridging and bending interactions is suggested.

## Introduction

Living organisms in all three kingdoms of life compact and organize their genomic DNA into nuclei or cells using various architectural proteins. Generally, the architectural proteins that modulate genome compaction function through DNA bending, bridging, wrapping and/or stiffening. These proteins share no sequence or structure similarities but appear to be functionally conserved across kingdoms. In addition, many architectural proteins are involved in important biological processes, such as transcription, replication, and repair [1–4]. In eukaryotes, histones wrap double-stranded DNA (dsDNA) into nucleosomes, the principal units of DNA compaction and organization [5,6]. The high-mobility group (HMG)-box proteins play an important architectural role in nucleoprotein complex assembly [7,8]. The nucleosome interacts with the linker histones H1 and H5, producing increasingly higher-ordered structures [9,10]. Bacterial nucleoid-associated proteins (NAPs), such as histone protein from strain U9 (HU) [11–13], integration host factor (IHF) [13,14], factor for inversion stimulation (Fis) [15,16] and histone-like nucleoid-structuring protein (H-NS) [17,18], contribute to the compaction of chromosomal DNA. Archaeal NAPs use strategies from both eukaryotes and bacteria. Most euryarchaea possess true histone proteins such as HMfA and HMfB from *M*. *fervidus* which organize dsDNA into tetrameric nucleosomes containing a tetramer of proteins that are analogous to the proteins in the eukaryotic [H3–H4]_2_ tetrasome [19–20]. Previous genome-wide MNase/ChIP-seq studies showed that archaeal histones HTkB and HMtA2 bind along the genome to form multimers of varied sizes in *T*. *kodakarensis* and *M*. *thermautotrophicus*, respectively [21–22]. In contrast, with few exceptions, crenarchaea do not synthesize histone homologues; instead, they use a wide array of small NAPs to package their DNA [23].

*Sulfolobus* species (e.g., *S*. *solfataricus* and *S*. *acidocaldarius*), which are hyperthermophilic crenarchaeal organisms, encode a variety of small, abundant and basic proteins ranging in molecular weight from 7 to 10 kDa that modulate their genome. Five classes of architectural proteins have been characterized and classified as histone-like proteins according to their physical properties: Alba (acetylation lowers binding affinity, also known as the Sso10b [24–26]/Sac10b [27] family), Sso10a [28]/Sac10a [29], Sul7d (Sso7d/Sac7d) [30–32], Cren7 [33,34] and Sso7c4 [35,36]. As observed in atomic force microscopy (AFM) images, Alba proteins form dimers in solution and exhibit two different binding modes by bridging DNA or forming stiff filaments along DNA, depending on the dimer concentration and composition [26]. In contrast, Sso10a also exists as a dimer in solution via antiparallel coiled-coil interactions. Sso10a1 bridges DNA duplexes, but Sso10a2 forms filaments along DNA [28]. Sul7d non-cooperatively binds to dsDNA as a monomer with micromolar affinity and introduces a sharp kink into the DNA duplex via the intercalation of hydrophobic side chains into the minor groove [30–32]. Cren7 is the only NAP known to be conserved at the kingdom level in crenarchaea. Cren7 is a structural homologue of Sul7d, and the biochemical properties and structures of both Cren7 and its DNA complex are similar to the properties and structures of Sul7d [33,34].

The Sso7c4 protein was originally identified in co-purifications with Sso7d and ribonuclease A from the supernatant of acid-soluble cell lysates of *S*. *solfataricus*. Based on the sequence alignment, Sso7c4 which has been classified as a bacterial repressor-like protein exhibited similarities with three transcriptional repressors, Ecrep 6.8 and Ecrep 7.3 in *E*. *coli*, and ACCR in *Agrobacterium tumefaciens* [35]. Sso7c4 appears to be the first repressor-like protein described in crenarchaea and may play a regulatory role in gene transcription [35]. The solution structure and DNA-binding properties of Sso7c4 have been determined by NMR analysis [36]. The Sso7c4 protein possesses a homodimeric DNA-binding fold that forms a swapped β-loop-β ‘Yin-Yang’ topology, and it belongs to the SpoVT-AbrB superfamily. The fold resembles the N-terminal DNA-binding domain of both AbrB and MazE, which are transcriptional regulators in bacteria [37,38].

Here, we present the crystal structure of wild-type Sso7c4, which was resolved at a high resolution of 1.63 Å, to obtain a better understanding of its architectural properties and functions. Two C-termini composed of LKEPWK residues, which are disordered in the crystal, are involved in binding DNA. Based on the results obtained from a variety of techniques, including tryptophan fluorescence quenching, fluorescence polarization (FP) and electron microscopy (EM), a model depicting the mechanism by which Sso7c4 binds DNA is proposed and further validated by fluorescence resonance energy transfer (FRET) analysis.

## Results and Discussion

### Overall structures of wild-type and C-terminally truncated Sso7c4

**Fig 1:**
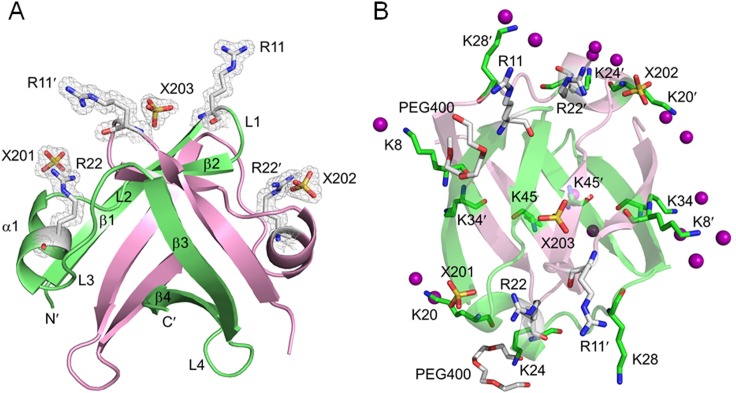
Crystal structure of the wild-type Sso7c4 protein. (A) Ribbon diagram of wild-type Sso7c4 with the two arginine pairs (R11/R22′ and R11′/R22) and three sulfates (X201, X202 and X203) depicted as balls and sticks with (2*F*o-*F*c) Fourier electron density maps (contoured at 1σ level). (B) Top view of the Sso7c4 dimer. The ribbon diagrams of the Sso7c4 dimer are colored green and pink to represent each monomer. All the lysine residues, the two arginine pairs, the three sulfates and the two PEG 400 molecules are depicted as balls and sticks. The water molecules surrounding the lysine side chains are drawn as purple spheres.

Sso7c4 crystals were grown in the presence of a 10-bp dsDNA to elucidate how the Sso7c4 protein interacts with dsDNA. However, crystals containing only proteins were formed. DNA appeared to be required for crystallization but was not incorporated into the crystal. The structures of wild-type and C-terminally truncated Sso7c4 (deletion of L49-K54) were well refined at resolutions of 1.63 and 1.4 Å, respectively (S1 Table). The crystal of wild-type Sso7c4 contains one homodimer in the asymmetric unit and is in the *P*2_1_2_1_2_1_ space group. Interestingly, the two basic C-termini are disordered owing to a lack of corresponding density. The wild-type Sso7c4 monomer has a β1-β2-α1-β3-β4 topology. The dimeric Sso7c4 protein, which consists of eight intertwined β sheets and two flanking α helices, forms a dimer via intermolecular associations between β1 and β3′ (the primes indicate the second monomer in the dimer) and β1′ and β3 in the flank, β2 and β2′ at the top, and β4 and β4′ at the bottom (Fig 1A). The crystal structure revealed that Sso7c4 displays a homodimeric DNA-binding fold, forming a swapped β-loop-β ‘Yin-Yang’ topology (Fig 1B). We also determined the crystal structure of C-terminally truncated Sso7c4 at a resolution of 1.4 Å to address the potential effect of the flexible C-terminus on DNA binding. In contrast to the wild-type structure, there are three homodimers in one asymmetric unit of the C-terminally truncated Sso7c4 crystal, which is in the *P*3_1_ space group (S1A and S1B Fig). The crystal structures of the wild-type and C-terminally truncated proteins are similar, as indicated by the good superposition of their α carbons (the r.m.s.d. value is 0.35 Å for 85 atoms, S1C Fig).

**Fig 2:**
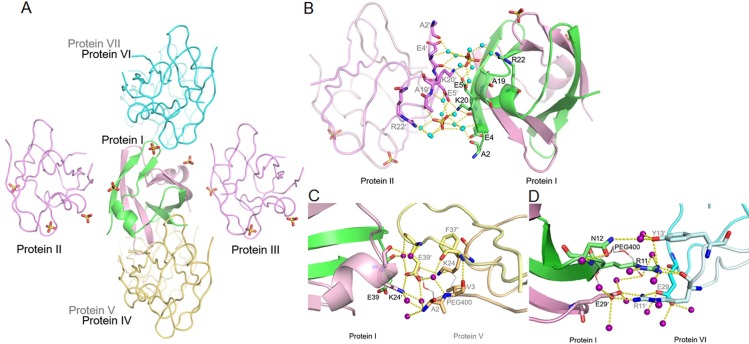
Protein-protein interactions in the wild-type Sso7c4 crystal. (A) One Sso7c4 protein interacts with six neighboring proteins through three patterns. Protein I is depicted as a ribbon and the six symmetry-related proteins are drawn as coils. (B) Detailed view of the sulfate-mediated interactions at the protein interface. (C) Detailed view of the electrostatic interactions between side chains of K24′ and E39′ at the protein interface. (D) Detailed view of the hydrogen bond arrays around the R11 (R11′) and E29 (E29′) side chains between proteins I and VI. All water molecules are displayed as cyan spheres in panel B and as purple spheres in panels C and D. The hydrogen bonds are shown as yellow dashed lines in all figures.

Transcription factors often contain an unusual distribution of positive charges owing to their specific function in binding DNA. Eight basic lysine residues are present in the Sso7c4 monomer: K8 in L1, K20 and K24 in α1, K28 in L3, K34 in β3, K45 in β4, and K50 and K54 in the C-terminus (Fig 1B). Most of these lysine side chains are dynamic, as shown by the larger B-factor and partially disordered map. However, K45 in β4 and K45′ in β4′ are well ordered in both the wild-type protein and the C-terminal truncation mutant because they are responsible for the intermolecular association of the Sso7c4 monomers, together with D38 (D38′), S40 (S40′) and E41 (E41′), through either electrostatic interactions or water-mediated hydrogen bonds at the dimerization interface (S2 Fig). In addition, K20 and K24 in the α1 helix participate in protein-protein crystal-packing contacts (Fig 2). Both K20 side chains of the wild-type protein are stabilized by electrostatic interactions with sulfates and E5′ side chains from the neighboring protein (symmetry-related molecules) in the crystal (Fig 2B). The K24 side chain is more ordered in chain A, which is stabilized by polyethylene glycol 400 (PEG 400) (Fig 1B), similar to the stabilization observed in crown ether binding mode [39]. On the other hand, the K24 side chain in chain B forms electrostatic interactions with the E39′ side chain of the neighboring protein (Fig 2C).

### Sulfates mediate protein-protein interactions in the wild-type crystal

In the wild-type crystal, Sso7c4 makes extensive contacts with neighboring molecules via residues in loop1 (R11, N12, and Y13), the α1 helix (K20, R22, and K24), loop3 (E29) and loop4 (E39). Consequently, their side-chain conformations are stabilized by protein-protein interactions. One Sso7c4 protein interacts with six neighboring proteins through three patterns: sulfate-mediated interactions with E5, K20, and R22, electrostatic interactions between K24′ and E39′, and hydrogen bond arrays around the R11 (R11′) and E29 (E29′) side chains (Fig 2). Three sulfate ions, which all bind to the top surface of wild-type Sso7c4, are present in the crystal structure. Two (X201 and X202) are located near the R22 (R22′) side chains, mediate protein-protein interactions, and are solvated by eight water molecules (Fig 1 and Fig 2B); the third (X203) is close to the β2′ strand on the DNA-binding surface (Fig 1 and S3 Fig). As shown in Fig 2B, the side chains of R22 and E5 in one monomer interact with sulfate X201 through water-mediated hydrogen bonds, whereas the K20′ side chain from the adjacent protein (symmetry-related molecule) makes direct contacts with the sulfate and E5 side chain via electrostatic interactions. The other sulfate, X202′, is located near the R22′ side chain of the symmetry-related protein and exhibits the same interaction patterns as sulfate X201. These two sulfates mediate interactions with the side chains of E5, K20 and R22 (E5′, K20′ and R22′ from symmetry-related protein) and are responsible for one of the three protein-protein interaction patterns at the interface. However, the third sulfate, X203, which is located on the top of the protein surface, is not involved in the protein-protein interaction. The strong direct ionic force surrounding this sulfate is provided by the NH groups of N12′ and Q14′ in one monomer and Q14 in the other monomer. The hydroxyl group of S10' forms a hydrogen bond with sulfate X203, which is only solvated by two water molecules (S3 Fig). We believe that the relative positions of these three sulfates on the top surface of Sso7c4 mimic the position of the phosphate backbone of DNA upon complex formation. As shown in Fig 2A, the serpentine trace of sulfates around the Sso7c4 surface through the crystal lattice suggested that Sso7c4 might bind DNA such that the DNA double helix is gently curved around the top surface of the protein dimer.

### Arginine pairs on the top surface of Sso7c4 are involved in binding DNA

The DNA-binding surface of Sso7c4, which was identified by NMR analysis, is composed of the highly dynamic loop1 (R11), as well as the β2 strand, α1 helix (R22) and loop3 [36]. Based on the sequence alignment of the Sso7c4 family in the archaeal kingdom, the only two arginines in Sso7c4, R11 and R22, are not only highly conserved across species in the archaeal homologues but are also involved in DNA contacts, as shown in previous NMR studies [36]. The R22 side chains in both wild-type and C-terminally truncated Sso7c4 are structurally conserved and more static than the R11 residues. In the crystal structure of the C-terminal truncation mutant, the R11 side chains of the three molecules in one asymmetric unit are all exposed to solvent and flexible (S1A and S1B Fig), consistent with the results of previous NMR dynamics studies [36]. In contrast, the R11 side chains in the wild-type Sso7c4 crystal structure are stabilized by intermolecular interactions. As shown in Fig 2D, the R11 side chain in protein I forms hydrogen bonds with the carbonyl oxygen of the R11′ backbone and the hydroxyl group of Y13′ from protein VI (symmetry-related molecule), but the R11′ side chain of protein VI forms polar contacts with the E29′ side chain of protein I. Similarly, the R11′ side chain of protein I interacts with the adjacent protein VII using the same interaction patterns as protein I and protein VI. In addition, the R11 side chain (chain A) of the wild-type protein is further stabilized by PEG 400 through water-mediated hydrogen bonds (Fig 2D).

**Fig 3:**
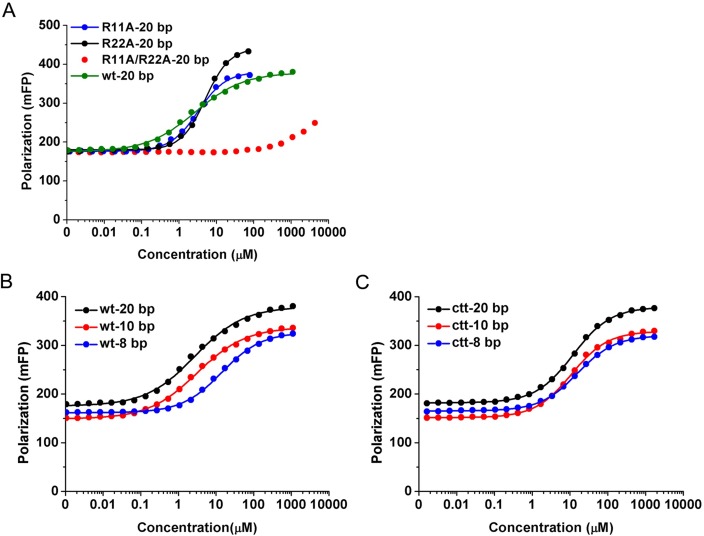
Sso7c4-DNA binding assay using FP. (A) Arginine mutant-DNA binding curves, (B) Wild-type Sso7c4-DNA binding curves, and (C) C-terminally truncated Sso7c4-DNA binding curves. The wild-type (wt)/C-terminally truncated (ctt) proteins were bound to 8-, 10- and 20-bp DNAs, and the three arginine mutants, Sso7c4-R11A, Sso7c4-R22A and Sso7c4-R11A/R22A, were bound to a 20-bp DNA in binding buffer. The graph depicts the average traces of three independent experiments for each sample, and the data points indicate the average values of technical replicates measured in triplicate.

In the wild-type crystal structure, both R22 side chains interact with sulfates, which are located near the α1 helices on the DNA-binding surface through water-mediated hydrogen bonds (Figs 1A and 2B). One Sso7c4 protein is associated with six symmetry-related molecules, of which four make contacts via R11 or R22 residues. Two of these neighboring molecules contact the R22 side chains via sulfate-mediated interactions (Fig 2B), whereas the other two interact with the R11 side chains via hydrogen bonds (Fig 2D). The relationship between R22 and the sulfate suggests that the positive side chain of R22 may interact with the negatively charged phosphates in the DNA backbone in the nucleoprotein complex. Similarly, R11 may contact the DNA base via hydrogen bonds. Based on these results, we speculate that the two arginines, R11 and R22′ (or R11′ and R22), may be involved in binding DNA. The R11 and R22 residues were replaced with alanine to probe their effects on DNA binding and further verify the roles of these two arginine pairs in Sso7c4. In FP binding assays (Fig 3A and Table 1), both single mutations, Sso7c4-R11A and Sso7c4-R22A, slightly decreased the binding affinity to a 20-bp dsDNA, d(CCAACACTGGCCAGTGTTGG)_2_. The binding affinities of Sso7c4-R11A (3.19±0.12 μM) and Sso7c4-R22A (5.07±0.15 μM) for a 20-bp DNA were weaker than that of the wild-type protein (2.73±0.18 μM). However, the Sso7c4-R11A/R22A double mutation significantly reduced the DNA-binding affinity. The double mutant cannot achieve a saturation binding curve, even at a very high protein concentrations (4.33 mM). Based on the estimated unsaturated DNA binding curve, the binding affinity of Sso7c4-R11A/R22A for a 20-bp DNA is much lower than that of the wild-type protein. Clearly, the two arginine pairs, R11, R22′ and R11′, R22, on the top surface of Sso7c4 are essential for binding DNA.

**Table 1:** Binding affinities for the arginine mutant/wild-type/C-terminally truncated/proteins in complex with three dsDNA fragments of different lengths.

DNA-binding affinity, Kd (μM)			
	8 bp	10 bp	20 bp
Sso7c4-R11A	NT	NT	3.19±0.12
Sso7c4-R22A	NT	NT	5.07±0.15
Sso7c4-R11A/R22A	NT	NT	NA
Wild-type Sso7c4	14.17±0.26	3.12±0.16	2.73±0.18
C-terminally truncated Sso7c4	15.90±0.53	10.99±0.41	12.01±0.53

NT: not tested, NA: not available.

The residues K8, R11, N12, Q14 and R22 of Sso7c4 are highly conserved in the archaeal homologues. In comparison with the N-terminal domain of AbrB (AbrB-N), which has a similar fold, the related DNA-binding residues D11, R15 and R23 in AbrB-N correspond to R11, Q14 and R22 in Sso7c4, respectively. The surface region of AbrB-N constituted by the conserved arginine residues (R8, R15, R23 and R24) has previously been identified essential for DNA binding [37]. As shown above, our results are consistent with the DNA binding property of AbrB-N. This finding suggests that Sso7c4 may bind to DNA in a similar way but prefer different DNA sequences than AbrB-N.

### The flexible C-termini of Sso7c4 are crucial for interactions with DNA

The role of flexible or dynamic protein segments in binding DNA has long been recognized. Most DNA-binding domains (DBDs) have N- or C-terminal extensions (arm or tail) that are rich in lysine and/or arginine and frequently disordered in solution [40]. The bacteriophage λ encodes two proteins, λ (cI) and Cro repressor, which play contrasting roles in regulating the growth mode of the phage. Both the λ and Cro repressors function as dimers, with the recognition helices of the HTH (helix-turn-helix) motifs inserted into the major grooves of the operator [41]. The HTH motif, in conjunction with the basic N-terminal arm (STKKKP), is responsible for the recognition and binding of λ repressor to the OL1 consensus operator half site. Lys3 and Lys4 contact guanine bases in the major groove, whereas Lys5 interacts with a phosphate group [42]. Deletion of the first six residues from λ repressor significantly reduces its binding affinity for the operator [43]. In contrast, the C-terminal residues of the Cro repressor (NKKTTA), some of which are disordered in the absence of DNA, form an extended arm that has been shown to be important for both specific and non-specific binding [44,45].

Interestingly, like the λ and Cro repressors, Sso7c4 also has a flexible and basic C-terminus (LKEPWK) that may participate in binding DNA. Here, we address the possible role of the Sso7c4 C-terminus in binding DNA using tryptophan fluorescence quenching, FP, and EM. The fluorescence emission spectrum of Sso7c4 was essentially the same as the spectrum expected for a free tryptophan, indicating that the single tryptophan (W53) located at the C-terminus is highly exposed to solvent in the Sso7c4 monomer. The binding of the wild-type Sso7c4 protein to dsDNA led to a dramatic decrease of nearly 98% in the quenching of the intrinsic tryptophan (W53) fluorescence (S4 Fig). Thus, the W53 residue at the Sso7c4 C-terminal end is involved in the interaction with DNA. A nonlinear fit of the titration data for Sso7c4 binding to a 20-bp dsDNA yielded a binding affinity of 22.77 μM (S4 Fig, right panel). Sso7c4 binds to dsDNA with micromolar affinity, consistent with other nonspecific dsDNA-binding proteins, such as Sso7d/Sac7d in *Sulfolobus* species, as shown in a tryptophan fluorescence quenching study [46].

The intensity of tryptophan fluorescence is proportional to the concentration of the Sso7c4 proteins in the micro-molar concentration range. Our gel-filtration results indicated that the Sso7c4 proteins are unable to form higher multimers in solution. The crystal structure of Sso7c4 indicated that the two basic C-termini in a dimer are highly flexible. In the above experiment, the Sso7c4 solution (~7 μM) was titrated with 20-bp dsDNA fragment, and the tryptophan fluorescence intensity of Sso7c4 decreased gradually to 98% quenching. Based on these results, the fluorescence quenching of tryptophan residue at the C-terminal end was more likely due to DNA binding rather than stacking of the tryptophan side chains between adjacent Sso7c4 proteins.

To further examine the role of its C-terminus for DNA binding, the residues L49 to K54 were truncated from Sso7c4. Then we used fluorescence polarization to study the binding of wild type Sso7c4 and the C-terminally truncated protein to 8-bp, 10-bp and 20-bp dsDNA fragments. The FP results showed that all the C-terminally truncated proteins exhibited lower binding affinity to 8-bp, 10-bp, and 20-bp DNAs than the wild-type protein (Fig 3B and 3C and Table 1). Undoubtedly, these FP data further show that the C-terminal ends of Sso7c4 participate in the interaction with DNA. However, the C-terminally truncated protein displayed a similar binding affinity (15.90±0.53 μM) for the 8-bp DNA to the wild-type protein (14.17±0.26 μM). The distances between the S atoms of X201 and X203 and between the S atoms of X202 and X203 were 13 Å and 16.1 Å, respectively (Fig 1). The sum of the distances of the three sulfates in the crystal was 29.1 Å, which corresponds to the length of an 8-bp DNA. When the 8-bp DNA interacted with the top DNA-binding surface of Sso7c4 at equilibrium, it would be too short to contact the C-terminus of the protein. Therefore, the wild-type Sso7c4/8-bp and C-terminally truncated Sso7c4/8-bp complexes had similar DNA-binding affinities. Extension of the DNA length to 10 bp would allow the flexible C-terminus to make contacts with the DNA, a scenario supported by the ~5-fold increase in the DNA-binding affinity of the wild-type Sso7c4/10-bp DNA complex (3.12±0.16 μM). Moreover, the binding affinity of the wild-type Sso7c4/20-bp DNA complex (2.73±0.18 μM) was slightly higher than but similar to the value for the wild-type Sso7c4/10-bp DNA complex, indicating that 10 bp is the shortest DNA that contacted by the two C-termini of Sso7c4. Thus, the length of the nucleotide molecule that can bind one protein dimer is at least 10 bp. In contrast, the C-terminally truncated protein bound to the 10-bp and 20-bp DNAs with similar affinities (~11 μM) but bound to the 8-bp DNA with slightly weaker affinity. Together, these results imply a mechanism for the formation of the complex: Sso7c4 first associates with DNA through its top surface, and the C-terminus at the base of the protein subsequently binds the DNA. The binding of the top surface of Sso7c4 to DNA accounts for the 10–15 μM binding affinity, but the binding affinity increases to ~3 μM when the C-terminus participates in the DNA interactions.

### Sso7c4 mediated DNA compaction, as visualized by EM

Electron microscopy is a suitable method for examining the sizes and shapes of biological macromolecules, particularly nucleoprotein complexes. The true nature of the complexes formed between Sso7c4 and DNA remained unclear; therefore, we performed a comparative EM study to investigate the changes in the DNA structure upon binding to Sso7c4. Here, we prepared EM samples using the cytochrome c spreading and rotary shadow casting with tungsten methods. The spreading of molecules to be adsorbed onto the support film in an extended and non-aggregated form is a key step in preparing EM samples. The incubation of negatively charged nucleic acids with a basic protein, cytochrome-c, is the most widely used method for spreading. The spread preparations are rotary shadowed with tungsten for contrast enhancement [47]. The individual proteins are bound to the DNA but cannot be distinguished in EM images due to their small sizes.

**Fig 4:**
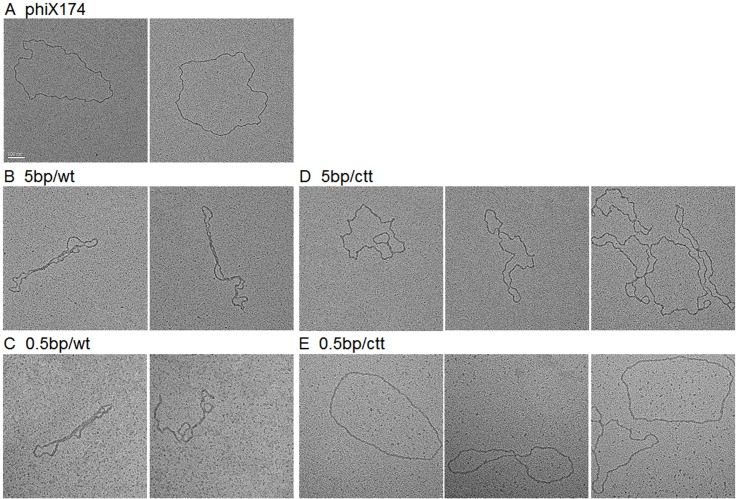
Electron micrographs of the phiX174 plasmid with or without the wild-type/C-terminally truncated Sso7c4 proteins. Representative images of Sso7c4-DNA complexes visualized by EM. Nicked phiX174 plasmids were incubated with different stoichiometries (5 bp/dimer or 0.5 bp/dimer) of either the wild-type (wt) or C-terminally truncated (ctt) proteins. (A) High magnification images of the relaxed, circular phiX174 plasmid alone. (B) and (C) High magnification images of the wild-type Sso7c4-plasmid complex. (D) and (E) High magnification images of the C-terminally truncated Sso7c4-plasmid complex. The scale bar represents 100 nm.

The effect of Sso7c4 binding to a relaxed, circular double-stranded phiX174 plasmid was examined at two different protein:DNA ratios: 1:5 or 1:0.5 protein dimer:DNA base-pair ratios. A series of representative images for each stoichiometry is shown in Fig 4 and the corresponding raw EM images are displayed in S5 Fig. The complexes were classified by visual inspection of the EM images as an ‘open’ or a ‘bridged’ form and quantified by different stoichiometries (S2 Table). Molecules with no crossings (Fig 4A and 4E, left) or single DNA crossings (Fig 4E, centre) were classified as an ‘open’ form. Molecules with bridged regions, of which the length is longer than a single crossover of two DNA duplexes, were classified as a ‘bridged’ form (Fig 4B, 4C and 4D).

In the absence of Sso7c4, the relaxed phiX174 plasmids displayed a circular form (Fig 4A). In the presence of the wild-type protein at two stoichiometries, Sso7c4 binding resulted in the formation of intra-molecular bridges in most regions, and the plasmid was heavily twisted, causing the DNA to condense (Fig 4B and 4C). At a ratio of 5 bp per dimer, most wild-type Sso7c4-DNA complexes are bridged (93%, n = 134), and the remaining open loops are primarily located at the ends of the plasmids. At increased wild-type Sso7c4-DNA ratios (0.5 bp/dimer), more bridged complexes were observed (98%, n = 86), of which some of the DNA molecules were completely bridged (15%, n = 86) (Fig 4C, right). These wild-type Sso7c4-DNA complexes are similar to the bridged protein-DNA complexes observed for the bacterial H-NS [17], the archaeal Sso10a1 [28] and Alba1 (Sso10b1) at low protein concentrations [26]. In contrast, the addition of comparable concentrations of C-terminally truncated Sso7c4 induced the formation of complexes that were distinctly different from the complexes observed with the wild-type protein. The plasmids bound to the C-terminally truncated proteins still retained a relaxed appearance and did not display a significantly twisted structure (Fig 4D and 4E). At a protein-to-DNA ratio of 1 dimer to 5 base pairs, binding of the C-terminally truncated Sso7c4 resulted in intra-molecular bridges as seen with the wild-type protein. The proportion of the plasmid bridged by C-terminally truncated Sso7c4 (70%, n = 178) is less than the wild-type (93%, n = 134). Furthermore, in a single plasmid, the number of bridged regions by C-terminally truncated Sso7c4 is much fewer than by the wild-type protein. At higher protein:DNA ratios (1:0.5 dimer:bp), a considerable number of C-terminally truncated Sso7c4-DNA complexes were observed to form a more open circular shape (67%, n = 109) (Fig 4E), suggesting that the C-terminally truncated proteins induced DNA stiffening. The round and open shape of the DNA molecules is a signature of DNA stiffening, as shown in previous studies on bacterial HU [12], archaeal Sac10a2 [28] and Alba1 proteins at high protein concentrations [26].

For a better understanding of the architectural properties of Sso7c4, the contour length and width of the naked, relaxed DNA molecules and the protein-DNA complexes were measured and are summarized in Table 2. The average length of the naked plasmid was 1844.72 nm, and the average width was 6.39 nm. The average length was in good agreement with the expected length of B-form DNA (5,386 bp, 5386×0.34 = 1831.24 nm), but the width was thicker than the expected value (~2 nm DNA diameter), because tungsten shadowing greatly exaggerates the thickness of the DNA molecules [47]. Two different nucleoprotein complexes that formed with either wild-type or C-terminally truncated Sso7c4 at a ratio of 5 bp per dimer were thicker than the plasmid duplex circle, with widths of 8.05 nm for the wild-type and 8.57 nm for the truncated. This increase in the width of the plasmid indicates that the DNA molecules were binding to proteins. On the other hand, the binding of wild-type Sso7c4 at a higher protein concentration (0.5 bp/dimer) increased the plasmid width up to a maximum of 13.89 nm, which was probably mediated by the extensive protein-protein interactions with unbound wild-type Sso7c4. Multimeric forms of Sso7c4 have not been observed in solution, suggesting that these protein-protein interactions may only be possible between Sso7c4 dimers that are bound to DNA.

**Table 2:** Average lengths and widths of the plasmid and nucleoprotein complexes. EM images were measured using ImageJ software.

Sample	Average length (nm) (n = 5)	Average width (nm) (n = 5)
PhiX174 plasmid	1844.72±5.71	6.39±0.28
Wild-type Sso7c4-plasmid (5 bp/dimer)	1643.33±9.46	8.05±0.22
Wild-type Sso7c4-plasmid (0.5 bp/dimer)	1494.58±20.41	13.89±0.25
C-terminally truncated Sso7c4-plasmid (5 bp/dimer)	1834.97±8.98	8.57±0.32
C-terminally truncated Sso7c4-plasmid (0.5 bp/dimer)	1830.06±8.81	9.16±0.20

n: the number of molecules.

Measurements of the contour lengths of the wild-type-DNA complexes at a ratio of 5 bp per dimer revealed a ~10.9% reduction in the mean length (from 1844 nm to 1643 nm) compared with that of a naked plasmid in the same preparation (Table 2). Upon incubation of the DNA with a ten-fold higher wild-type protein concentration (a ratio of 0.5 bp per dimer), the average contour length was as short as 1494.58 nm, which corresponds to a ~19.0% reduction in length due to DNA bending. In contrast, little or no condensation of the DNA molecules was observed in the C-terminally truncated Sso7c4-bound complexes at stoichiometries of 5 bp and 0.5 bp DNA per dimer. Although the individual protein cannot be resolved, a reduction in apparent DNA length might be attributed to DNA bending, which in turn can be induced by binding to wild-type Sso7c4. However, the DNA contour length was not reduced upon C-terminally truncated Sso7c4 binding (1834.97 nm for 5 bp/dimer and 1830.06 nm for 0.5 bp/dimer), implying that both C-termini of Sso7c4 are also involved in bending DNA. Interestingly, based on the results of the FP binding assay and EM images, the basic and flexible C-termini of Sso7c4 are responsible for binding DNA and participate in bending DNA. In conclusion, wild-type Sso7c4 induces the formation of a more compact DNA structure primarily through both bridging and bending interactions, whereas C-terminally truncated proteins rigidify and open the DNA molecules at high protein concentrations.

### Proposed Sso7c4-DNA model and validation using FRET

**Fig 5:**
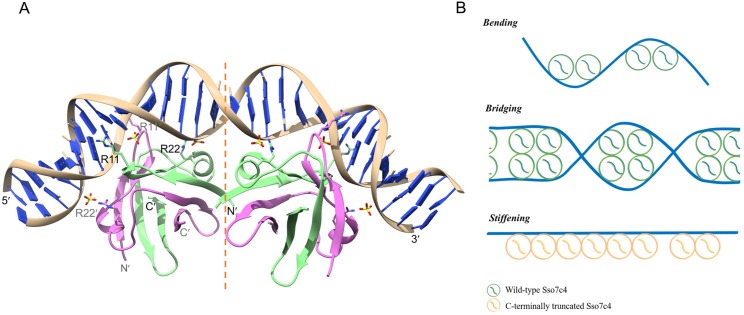
Model of the interaction of Sso7c4 with DNA. (A)The DNA-binding surface of the Sso7c4 dimer was manually docked into the major groove such that the sulfates were located as close as possible to the phosphates in the DNA backbone. A symmetrically bent DNA conformation was constructed using a known curved DNA (from the CAP-DNA complex; 1O3T). A brief minimization was performed to remove steric hindrance between the protein side chains and DNA. (B) The models of three DNA binding modes induced by Sso7c4 variants. The wild-type Sso7c4 binds to DNA in two different modes, bending and bridging. In contrast, the C-terminally truncated Sso7c4 binds side-by-side along the DNA molecule leading to DNA stiffening.

Three sulfates are bound to the top DNA-binding surface of Sso7c4 in the crystal lattice. The curved trace of these sulfates mimics the position of the phosphate backbone of DNA. Furthermore, the analysis of the EM images of the wild-type-DNA complexes implied that wild-type Sso7c4 induces DNA bending. In addition, Sso7c4 has been classified as a repressor-like protein by the amino acid sequence alignment [35]. A previous NMR study of DNA titrated with the protein indicated that Sso7c4 interacts with DNA in the major groove [36]. In our FP assay, 10 bp of DNA is the shortest length that binds to one Sso7c4 dimer. Based on these results, we propose a possible model for the Sso7c4-DNA complex. We searched all possible structures of protein-DNA complexes whose proteins act as transcriptional regulators by bending DNA as candidates for constructing our model. We attempted to fit the positions of three sulfates as closely as possible to the phosphates in the bent DNA molecule. Surprisingly, these three sulfates fit well with the phosphates of curved DNA molecules in the CAP (catabolite activator protein)-DNA complexes (PDB ID: 1O3T) [48]. The Sso7c4-DNA model shown in Fig 5A is approximately 2-fold symmetric, with each Sso7c4 dimer interacting with one half-site of symmetry-related DNA. Two swapped-β-loop-β motifs on the top of Sso7c4 contact the major groove on the concave side of the curved DNA. Based on our studies, we conclude that the Sso7c4 proteins function as chromosomal proteins which bind DNA with micro-molar affinity and display different architectural properties. The models of DNA bending, bridging and stiffening induced by Sso7c4 variants were shown in Fig 5B.

FRET is a useful tool for the precise determination of the DNA deformation caused by proteins because small changes in the distance between two fluorophores (typical 2–10 nm) lead to large changes in energy transfer [49,50]. Here, we validated the proposed model of the Sso7c4-DNA complex using bulk FRET measurements. Solution-based FRET assays were performed on the 20-bp, 24-bp and 30-bp dsDNAs at saturating protein concentrations (S6 Fig). Therefore, we determined the end-to-end distance of the DNA in the absence or presence of protein by measuring the change in FRET efficiency (*E*_*FRET*_) between two cyanine dye molecules covalently attached to the 5'-ends of the target DNA duplex. Protein-induced DNA bending decreases the end-to-end distance of the duplex and results in an increase in the FRET efficiency between two dye molecules. The average FRET efficiency and end-to-end distance of DNA are shown in Table 3.

**Table 3:** FRET efficiency and distance of the DNA duplex alone or bound to Sso7c4 in bulk FRET experiments.

	DNA alone		Wild-type Sso7c4-DNA		C-terminally truncated Sso7c4-DNA	
	E_FRET_	Distance (Å)	E_FRET_	Distance (Å)	E_FRET_	Distance (Å)
20 bp	0.1999±0.0060	67.42±0.42 (68.0)^a^	0.2159±0.0034	66.33±0.22	0.2059±0.0044	67.30±0.30
24 bp	0.0747±0.0012	81.38±0.24 (81.6)^a^	0.1041±0.0017	76.59±0.23 (76.6)^b^	0.0807±0.0018	80.25±0.32
30 bp	0.0397±0.0010	90.98±0.39 (102.0)^a^	0.0513±0.0003	86.99±0.08 (86.8)^b^	0.0292±0.0005	95.92±0.30

^a^Theoretical value based on B-DNA

^b^Distance measured from the proposed model.

Using a 24-bp DNA duplex, the FRET efficiency was 0.0747 for free DNA; after the addition of the protein, *E*_*FRET*_ increased to 0.104 for wild-type Sso7c4 and 0.0807 for the C-terminally truncated mutant. Based on these values, we calculated end-to-end DNA distances of 81.38 Å for free DNA, 76.59 Å in the presence of wild-type protein, and 80.25 Å in the presence of C-terminally truncated Sso7c4. Compared with the unbound DNA, the dye-to-dye distance in the wild-type complex was decreased, whereas the binding of C-terminally truncated Sso7c4 produced little difference in distance (Table 3). The decreased distance upon complex formation indicates that wild-type Sso7c4 bends the DNA, as observed in the EM analysis (Table 2). In contrast, the binding of C-terminally truncated Sso7c4 induced a slight change in the end-to-end distance of DNA, consistent with the length measured from the EM images of C-terminally truncated Sso7c4-DNA complexes. The FRET data confirm the EM observation showing that the C-terminally truncated protein forms a rigid filament along DNA (Fig 5B).

The end-to-end distance of free DNA (20 and 24 bp) determined by FRET is in good agreement with the theoretical value for canonical B-DNA (Table 3). However, the measured distance is shorter than the expected value for the 30-bp free DNA. The sequence of the 30-bp DNA used in the FRET experiments contains “ATAA”, which may have an intrinsic sequence-directed curvature, resulting in the decreased distance compared with the distance for canonical B-DNA. In the presence of wild-type Sso7c4, the end-to-end distance of the 30-bp DNA decreased to 86.99 Å. Clearly, the binding of wild-type Sso7c4 results in DNA bending. Interestingly, the binding of the 30-bp DNA to the C-terminally truncated protein exhibited a larger end-to-end distance than free DNA (95.92 Å for the C-terminally truncated-DNA complex and 90.98 Å for 30-bp DNA). The binding of C-terminally truncated Sso7c4 seems to straighten the DNA and produces a stiffened configuration, as shown in the EM images.

On the other hand, the end-to-end distance of the 20-bp DNA only changed slightly upon binding to either the wild-type or C-terminally truncated protein compared with the distances for free DNA. Both the 24- and 30-bp DNAs, but not 20-bp DNA, were bent by wild-type Sso7c4. In our model, ten DNA base pairs are covered by one Sso7c4 dimer. Thus, the entire 20-bp DNA binds to two Sso7c4 dimers located side by side at saturating protein concentrations. However, the 20-bp DNA appears to be too short to observe the reduction in the end-to-end distance caused by the binding of wild-type Sso7c4. The end-to-end distance of the 20-bp DNA also remains constant upon C-terminally truncated Sso7c4 binding, consistent with the results for the 24- and 30-bp DNAs, suggesting stiffening of the DNA through side-by-side binding along the DNA molecule.

The end-to-end distances of the 24- and 30-bp DNAs observed upon binding to wild-type Sso7c4 are almost identical to the corresponding distances measured from our proposed model. The FRET results support the fidelity of the proposed model. In addition, the end-to-end distance of the DNA in the model is 86.8 Å, which corresponds to a ~14.7% reduction in length compared with the length for 30-bp B-DNA. This reduction in the length of the DNA upon binding to Sso7c4 is consistent with the EM results, in which the DNA length was reduced by 10.9% - 19.0% at stoichiometries of 5 bp and 0.5 bp DNA per dimer. The roles of the Sso7c4 C-terminus in both binding and bending DNA have been revealed *in vitro* using EM and FRET analysis. Interestingly, the HMG-box tails (C-terminal for HMG-D and LEF-1, N-terminal for yeast NHP6A) show a similar property, as the loss of extended tails leads to a substantially reduced DNA affinity and a smaller bend angle [51].

### Biological implications

DNA-binding proteins that compact and regulate genetic material are crucial in all organisms. In *Sulfolobus* species, NAPs predominantly organize the genome by bridging or bending DNA. The Sso10b and Sso10a proteins have been shown to bridge or rigidify DNA in EM [52] and AFM studies [26,28], whereas Sac7d/Sso7d [30–32] and Cren7 [33,34] were shown to bend DNA in their complex 3D structures. In our study, Sso7c4 exhibits DNA-binding activity and alters the trajectory of the DNA through bending and bridging. Sso7c4 might be a transcriptional regulator, based on its amino acid sequence and our proposed model. Sso7c4 utilizes two swapped-β-loop-β motifs for binding DNA, which is similar to the fold of AbrB-N [37,38] but distinct from the HTH motif, a common recognition element used by most prokaryotic transcriptional regulators. However, our proposed model of the Sso7c4-DNA complex resembles many structures of transcriptional regulators, such as CAP [48,52–54] and Fis [16] from *E*. *coli*, as well as λ [41,42] and Cro repressor [41,44] from bacteriophage lambda, in complex with DNA. These regulators use an HTH motif to bind the major groove of DNA and function as a homodimer, with each motif binding one half-site of the symmetry-related DNA. The binding of all these transcriptional regulators bends DNA to various degrees. Based on a structural analysis, amino acid residues on the second α-helix (recognition helix) of this motif are the most important residues for protein-DNA recognition.

In our model, two Sso7c4 dimers form symmetric contacts with the individual major grooves of DNA. The side chain E5 in one Sso7c4 contacts the K20 residue in the other Sso7c4 and vice versa through electrostatic interactions at the protein-protein interface. Interestingly, similar protein-protein interaction patterns between K20 and E5 are also observed in the crystal lattice contacts (Fig 2B). Most of the conserved crystal lattice contacts involve residues near the surfaces of wild-type Sso7c4 that form protein-protein interactions or protein-DNA contacts in our proposed model. In the Sso7c4 crystal structure, the side chains of Ser10′, Asn12′, Gln14′ and Gln14 form contacts with sulfate X203 (S3 Fig). In the Sso7c4-DNA model, the side chains of Ser10′, Asn12′, Thr16′ and Gln14 on the DNA-binding surface interact with phosphates in the DNA backbone. The R11 residue in the flexible loop L1 contacts a DNA base in the major groove, whereas the R11′ side chain is located near the minor groove on the opposite site of the DNA. Interestingly, many transcriptional regulators utilize similar residues (Arg, Ser/Thr, Asn and Gln) in the recognition helix of the HTH motif to contact the major groove of their operators. The first two residues in the recognition helix of both Cro and λ repressors, Gln27 and Ser28 in Cro and Gln44 and Ser45 in λ repressor, form similar base contacts with invariant bps 2 and 4 of all operator half-sites [41,42,44]. In addition, the Asn31 in the recognition helix of Cro repressor interacts with a phosphate in the DNA backbone [44]. The Asn52 and Asn55 residues are located in the loop following the recognition helix of λ repressor: Asn52 interacts with backbone phosphates, and Asn55 contacts a DNA base within the major groove [42]. In the CAP-DNA complex, Arg180, Glu181 and Arg185 of the recognition helix form H bonds with bases lining the DNA major groove, and Ser179 and Thr182 interact with the phosphates of the DNA backbone [53,54]. In the Fis-DNA complex, the three side chains of Asn84, Thr87, and Lys90 stabilize the interaction of the recognition helix with the DNA backbone, and only two residues, Arg85 and Asn84, from each recognition helix contact bases within the major groove by H bonds [16].

The extended N- or C-terminal tails of DNA-binding domains often increase the affinity for the target DNA and thus represent crucial segments for gene activation or repression [39]. Based on the FP, EM, and FRET analyses, the basic and flexible C-termini of Sso7c4 may act as two hands that grasp the DNA molecule, further inducing DNA bending. In our model, the last five residues of each monomer are not shown because they are disordered in the Sso7c4 structure, but the C-termini in two of four monomers (colored in magenta in Fig 5A) may form contacts in the vicinity of the DNA backbone in the middle region of the DNA, as shown in the Cro-DNA complex [44].

Based on the substantial similarity of the model of the DNA complex to transcriptional regulators, we assume that Sso7c4 has a regulatory role in gene transcription in archaea. As shown in our EM analysis, the binding of wild-type Sso7c4 bends and bridges DNA. In general, transcriptional regulators modulate transcription either by directly interacting with RNA polymerase or by forming DNA bridging structures. For example, λ repressor contains a helix-turn-helix DNA-binding domain and a small surface patch that contacts RNA polymerase for transcriptional activation [41,42]. The CAP-cAMP complex assists RNA polymerase in binding to the promoter, thereby stimulating catabolic operon transcription [55]. Fis functions not only by interacting with effectors like RNA polymerase, but also by bending DNA [16]. On the other hand, bridge formation is also important for transcription, as the bridges can potentially trap or exclude RNA polymerase from promoters. Consequently, by bridging and bending DNA, Sso7c4 may have implications on both nucleoid structure and gene regulation.

Living organisms in all three domains rely on DNA to store and replicate their genetic information, despite the differences in their cellular organization [1–4]. In eukaryotes, the major architectural proteins are histones, which wrap DNA and form nucleosomes. The eukaryotic histones bind and wrap DNA using conserved residues, including the arginine and lysine residues of the α1 helix, an arginine in L1, and a lysine and threonine in L2 that directly participate in DNA binding. When examined collectively over all 12 histone fold DNA-binding sites, the arginine side chains most closely associated with the minor groove show variable contacts with the surrounding DNA and protein [5,6]. Coincidentally, arginine residues R11 and R22 of Sso7c4 are also essential for binding DNA. In our model, two R22 residues from one Sso7c4 dimer contact the DNA backbone through electrostatic interactions; in addition, R11 forms contacts with DNA bases in the major groove, and R11′ is located near the minor groove. Interestingly, superposition of the conserved R22 side chain in Sso7c4 with R72 of the α1 helix in histone H3 (1KX5) reveals that the R11′ side chain on the DNA-binding surface of Sso7c4 is located in the DNA minor groove, similar to R83 in loop1 of H3 (S7A Fig). We chose R22 of Sso7c4 and R72 of histone H3 for alignment because they are both located on the surface of the α-helices and participate in contacts with the DNA backbone. Therefore, one arginine pair in the Sso7c4, R11' and R22, may bind DNA similar to histones in eukaryotes.

On the other hand, the archaeal histones HMfA and HMfB from *M*. *fervidus* share sequence homology with the eukaryotic nucleosome core histones H2A, H2B, H3 and H4. In comparison with histone H3, which has a similar fold, the related DNA-binding residues R72 and R83 in H3 correspond to R11 and R20 in HMfA (as well as the equivalent residues in HMfB), respectively. When the crystal structures of HMfA (PDB 1B67) and H3 (H3 histone in the nucleosome, 1KX5) are superposed by using the α carbons, the orientation of R11 side chain in HMfA is quite different from that of R72 in H3 (S7B Fig). Therefore, the similarity between R22 of Sso7c4 and R72 of H3 is not readily observed in R11 of HMfA. Nevertheless, it is likely that the R11 side-chain conformation of HMfA could be changed, similar to that of R72 in H3, upon DNA complex formation. Furthermore, based on the analysis our EM images, Sso7c4 might package DNA through a bending and bridging mechanism. A similar mechanism for DNA compaction has been observed for the eukaryotic histone linker H1/H5 [9,10], bacterial nucleoid-associated protein Fis [15,16] and tetrameric LrpC (leucine-responsive regulatory protein) from *Bacillus subtilis* [56]. These proteins exhibit very different global folds but use similar strategies for DNA packaging. We believe that the chromatin in bacteria and archaea is organized and compacted through similar mechanisms as eukaryotes. Future investigations of the exact binding mode of Sso7c4 in a complex with DNA will lead to a better understanding of chromosomal organization and gene regulation.

## Materials and Methods

### Gene cloning and protein overexpression and purification

The gene encoding the DNA-binding protein Sso7c4 was amplified from the *S*. *solfataricus* P2 genome using PCR and cloned into the pET-29a vector using the NdeI and BamHI restriction sites. Sso7c4 was overexpressed in *E*. *coli* strain BL21 (DE3) codon plus-RIL following induction with 0.4 mM IPTG in LB medium containing kanamycin (50 μg/ml) and chloramphenicol (30 μg/ml) for 4 h at 37°C until an OD_600_ of 0.8–0.95 was attained.

The cells were lysed in binding buffer (20 mM Tris-Cl, pH 7.5) by sonication, and the lysate was incubated at 65°C for 30 min to remove most of the host proteins. The supernatants were purified on an SP cation-exchange chromatography FF column (GE Healthcare Life Sciences). The protein was eluted in binding buffer containing 200 mM NaCl. The fractions containing the Sso7c4 proteins were pooled, concentrated and further purified using a 16/60 Superdex-75 gel filtration column (GE Healthcare Life Sciences) equilibrated with binding buffer. The C-terminally truncated Sso7c4 (deletion of L49-K54) was constructed, overexpressed and purified using the same procedure as the wild-type protein. Site-directed mutagenesis was used to prepare the Sso7c4-R11A, Sso7c4-R22A, and Sso7c4 R11A/R22A mutants. The arginine mutants were prepared using the same method as wild-type Sso7c4, except that the protein was eluted with 50 mM NaCl. The selenomethionine (Se-Met) derivative of the recombinant Sso7c4-V21M protein was overexpressed using the Overnight Express™ Autoinduction System 2 (Novagen) containing 25 mg/l L-selenomethionine and purified as described above.

Protein concentrations were determined by measuring the ultraviolet absorbance at 280 nm using an extinction coefficient ε_280_ of 6990 M^-1^ cm^-1^ for wild-type Sso7c4 and the three arginine mutants and 1490 M^-1^ cm^-1^ for C-terminally truncated Sso7c4.

### Crystallization and X-ray diffraction data collection

Crystals of Sso7c4 were prepared at room temperature using the sitting-drop vapor diffusion method. The initial crystals were obtained by robotic screening. The crystals used for data collection were produced under optimized conditions consisting of equal volumes (typically 2 + 2 μl) of the protein solution in 20 mM Tris-Cl, pH 7.5, and their reservoir solutions. We initially attempted to obtain the crystal of the Sso7c4-dsDNA complex by mixing wild-type Sso7c4 and the 10-bp DNA (CCTATATAGG)_2_ at the same concentration (2 mM) and screened for crystallization. However, the crystals only contained the wild-type Sso7c4 proteins and not the DNA molecules. The reservoir solution for the wild-type Sso7c4 crystal consisted of 0.1 M Tris-Cl, pH 8.4, 40% PEG 400 and 0.2 M Li_2_SO_4_. In contrast, crystals of C-terminally truncated Sso7c4 and Sso7c4-V21M were obtained at a protein concentration of 60 mg/ml. The mother liquor for the C-terminally truncated Sso7c4 crystal consisted of 0.1 M Tris-Cl, pH 7.5, and 40% PEG 400, and the mother liquor for the Sso7c4-V21M crystal consisted of 0.1 M imidazole, pH 7.5, and 40% PEG 400.

All crystals were cryoprotected by soaking them in a solution consisting of the well solution supplemented with 25% PEG 400. The crystals were loop mounted and flash-frozen in liquid nitrogen for data collection. The X-ray diffraction data were collected at 123 K and processed using the HKL2000 package [57]. The synchrotron data were collected at the SPXF beamlines BL13B1 and BL13C1 at NSRRC (Taiwan) using an ADSC Q315r CCD detector. The in-house data were collected using the FR-E+ Super Bright/R-AXIS HTC image-plate detector system.

### Structure determination and refinement

Although a solution structure of Sso7c4 (PDB ID: 2L66) was previously determined by NMR [36], it did not yield a correct molecular replacement (MR) solution for the crystals. The structure was instead determined using the two-wavelength anomalous dispersion data collected from a crystal of a mutant protein in which Val21 was replaced with Se-Met. The phases of the crystals of the selenomethionine-derived Sso7c4-V21M protein were calculated using the 2.1 Å resolution data from two-wavelength anomalous dispersion experiments collected at a selenium high remote wavelength of 0.9635 Å and an inflection wavelength of 0.9789 Å. The locations of the two selenium sites were identified using the program SHELX/C/D/E [58], with an occupancy of greater than 0.8. The initial phases were solved using the program CRANK [59] of the CCP4 program suite [60], resulting in an overall figure of merit (FOM) of 0.497 for the 30.0–2.0 Å resolution data. The phases were further improved by modifying the density using the program DM [61], resulting in an FOM of 0.76 for the 24.40–2.45 Å resolution data. An initial model containing approximately 101 residues of two Sso7c4 molecules (homodimer) was automatically generated through alternating cycles of the Buccaneer program [62]. Additional residues were manually modeled in Coot [63] and refined by Refmac5 [64].

The phases for the wild-type and C-terminally truncated crystals were obtained via MR using the Phaser program [65]. The coordinates of Se-Met Sso7c4-V21M were used as a search model. The structural model was manually constructed in Coot and refined by restrained refinement with Refmac5. The difference Fourier (Fo-Fc) maps were calculated to locate the solvent molecules and ligands. Refinements were performed with native data using the Refmac5 program in CCP4, and the *R*_free_ calculations were performed on 5% of the reflections. All of the atoms in the protein were restrained in a similar manner using the restraints provided by the CCP4 refinement program and the automatically generated, local, non-crystallographic symmetry (NCS) restraints. The stereochemistry of the refined structure was validated using MolProbity [66]. All φ/ψ angles and other conformational parameters in these structures were within the acceptable regions. The crystallographic and refinement parameters for each structure are shown in S1 Table. The atomic coordinates and structural factors of the crystal structures presented in this study have been deposited in the Protein Data Bank (accession codes in S1 Table). All structures were produced using PyMOL (The PyMOL Molecular Graphics System, Version 1.7.6.4, Schrodinger, LLC).

### Oligonucleotides used in the FP assays

Synthetic DNA oligonucleotides were purchased from PURIGO Biotechnology Co., Ltd., Taiwan and purified by HPLC. The 20-bp self-complementary DNA was used for the fluorescence quenching study. In addition, the DNA was labeled on the 5′ end with an FAM fluorophore for the fluorescence polarization study. The DNA sequences were:

20-bp DNA: 5′-CCAACACTGGCCAGTGTTGG-3′, ε_260_ = 186500 M^-1^cm^-1^.

5′-FAM-20-bp DNA: 5′-FAM- CCAACACTGGCCAGTGTTGG-3′, ε_260_ = 207460 M^-1^cm^-1^.

5′-FAM-10-bp DNA: 5′-FAM-CCTACGTAGG-3′, ε_260_ = 117360 M^-1^cm^-1^.

5′-FAM-8-bp DNA: 5′-FAM-CGCTAGCG-3′, ε_260_ = 93860 M^-1^cm^-1^.

The dsDNA was prepared by dissolving the self-complementary oligonucleotides in binding buffer, heating to 90°C for 5 min, and then slowly cooling to 20°C at a rate of 0.02°C per second. All the binding assays in this study using the Sso7c4 protein and DNA were performed in binding buffer (20 mM Tris-Cl, pH 7.5).

### Tryptophan fluorescence quenching assay

Fluorescence titration was measured on a HITACHI 4500 Fluorescence Spectrophotometer with excitation at 295 nm (4 nm slit width) and emission monitored at 300–500 nm (8 nm slit width). The quenching of the tryptophan (W53) fluorescence in wild-type Sso7c4 upon binding to the 20-bp DNA d(CCAACACTGGCCAGTGTTGG)_2_ was performed in a 4 ml quartz cuvette with a 1-cm optical path length in binding buffer at 25°C by the stepwise addition of aliquots (e.g., 1 μl) of 5.4 mM dsDNA stock solutions to 2 ml of the wild-type Sso7c4 protein solution (7.0 μM homodimer). The binding of Sso7c4 to the dsDNA was indicated by the quenching of the tryptophan (W53) fluorescence (345 nm) in the protein as a function of the DNA concentration. The data were fit using nonlinear regression, assuming non-cooperative binding, to obtain the binding constant.

### Fluorescence polarization

The affinity of a protein-DNA interaction can be determined by monitoring the changes in FP associated with the formation of the complex under equilibrium conditions [67]. We used FP to study the equilibrium binding between the Sso7c4 proteins (wild-type, C-terminally truncated, Sso7c4-R11A, Sso7c4-R22A and Sso7c4-R11A/R22A) and 5′ FAM-labeled dsDNAs of different lengths. In a basic FP experiment, the protein was serially diluted in binding buffer (each sample with a volume of 50 μl) in the wells of a 96-well microplate (NUNC). Then, the 5′ FAM-labeled dsDNA was added to each well (50 μl of a 200 nM stock) to a final DNA concentration of 100 nM and a total binding reaction volume of 100 μl. In the binding assay using the Sso7c4-R11A/R22A mutant and 20-bp dsDNA, the mutant protein was serially diluted from a higher concentration of 4.33 mM to confirm its much weaker binding affinity. The binding reactions were incubated at room temperature for 1 h. After the binding reaction had reached equilibrium, triplicate reactions for each sample were measured at 25°C using the SpectraMax Paradigm plate reader (Molecular Devices, CA, USA) with an excitation wavelength of 485 nm and an emission wavelength of 535 nm. The equilibrium binding data were analyzed and plotted using Origin Pro8.

### Electron microscopy

The phiX174 RF II DNA (New England Biolabs) is the double-stranded, nicked, circular form of the phiX174 DNA. The molecular weight of phiX174 DNA is 3.50×10^6^ daltons, and it is 5,386 bp in length. A 1 mg/ml solution of phiX174 RF II DNA was diluted 20-fold into the binding buffer to a final concentration of ~15 nM (50 μg/ml). Complexes of protein and phiX74 were formed by incubating equal volumes of ~15 nM phiX174 DNA with 15 μM or 150 μM protein dimer solutions to achieve stoichiometries of 5 bp and 0.5 bp DNA per protein dimer in binding buffer for 30 min at 25°C.

The EM samples were prepared by the aqueous drop spreading and heavy metal shadowing methods [47,68]. Briefly, a solution of the DNA plasmid alone or Sso7c4-DNA complex was mixed with cytochrome c solution in 0.25 M ammonium acetate and incubated for 5 min. A 50-μl drop was placed on a clean Parafilm surface, and a thin carbon foil-covered grid was touched to the drop, washed with a water/ethanol series, air-dried, and subjected to rotary shadow casting with tungsten. The EM images were recorded at 200 kV on a Tecnai F20 Bio TWIN (FEI Co., Netherlands) using a slow-scan CCD camera (Gatan UltraScan 4000 4k x 4k, USA). The analysis of the EM images provides quantitative information about macromolecular assemblies containing DNA. The average lengths and widths of five randomly selected plasmid-nucleoprotein complexes were measured using ImageJ software [69].

### Molecular docking model of the Sso7c4-DNA complex

We used the chimera to build the complex model using a bent dsDNA from the CAP-DNA complex structure (1OT3). The DNA-binding surface of the Sso7c4 dimer was manually docked into the major groove to position the sulfates as close as possible to the phosphates in the DNA backbone. We also carefully considered sharp complementarity, avoided steric hindrance and maintained favorable electrostatics at the protein-DNA interface. Finally, we performed energy minimization with CHARMM27 [70] force-field parameters to remove steric hindrance between the protein side chains and DNA.

### Oligonucleotides used in the bulk FRET study

FRET efficiency is most sensitive to changes in distance when the separation between the donor and acceptor pair is near the Förster distance R_0_ (~53.5 Å, for our system).

The oligonucleotides used in the bulk FRET studies were:

20 bp_F DNA: 5′-CGT GCT CTG CGA TCT CTT CG-3′

20 bp_R: 5′- CGA AGA GAT CGC AGA GCA CG -3′

24 bp_F: 5′-CGT GCT CTG CGA TCT CTT CGA TAA-3′

24 bp_R: 5′- TTA TCG AAG AGA TCG CAG AGC ACG -3′

30 bp_F: 5′-CGT GCT CTG CGA TCT CTT CGA TAA CCA TAG-3′

30 bp_R: 5′- CTA TGG TTA TCG AAG AGA TCG CAG AGC ACG -3′

Strand F was labeled at the 5′ terminus with a Cy5 (acceptor) fluorophore. Strand R was labeled at the 5′ terminus with a Cy3 (donor) fluorophore.

Both labeled and unlabeled oligonucleotides were synthesized and HPLC-purified by PURIGO Biotechnology Co., Ltd., Taiwan. We calculated the DNA concentrations and dye labeling efficiencies by measuring the absorption in UV-VIS spectra from 200 to 750 nm (ε _Cy3, 550 nm_ = 150000 M^-1^ cm^-1^, ε _Cy5, 650 nm_ = 250000 M^-1^ cm^-1^). The dsDNA was prepared by mixing the two complementary oligonucleotides in equimolar amounts in assay buffer (20 mM Tris-Cl pH 7.5) to a final DNA concentration of 1.0 μM, heating to 90°C for 5 min, and then slowly cooling to 20°C at a rate of 0.02°C per second. The DNA sequences used in our studies, for either FP or FRET, were selected randomly because Sso7c4 binds to DNA without sequence specificity. The program OligoAnalyzer 3.1 (Integrated DNA Technologies) was used to analyze the DNA sequence to avoid the formation of hairpin structure. The GC contents of the DNA molecules were also calculated to make sure that all DNA molecules can form complete double-stranded DNA fragments at 25°C.

### Bulk FRET measurement

We prepared the Sso7c4-DNA complexes by incubating the dsDNA (1.0 μM) with 12.5 μM wild-type Sso7c4 or the C-terminally truncated mutant in assay buffer for 1 h at 25°C. Twenty microliters of each reaction were then added to individual wells of a 384-well borosilicate microplate (NUNC) and scanned with a Typhoon Imager 9400 (GE Healthcare). For all scans, the focal point was set to +3 mm from the surface, and light emission was collected with a photomultiplier tube (PMT) set to 450 V (sample scans in S5 Fig**)**. In each plate, fifteen wells contained buffer only and were used for background subtraction. Data processing and quantitation were performed using the Image Quant TL 5.2 software.

### FRET efficiency calculations

The bulk FRET efficiencies (E) were determined and calculated as previously described [71]. The FRET efficiency (E) from donor to acceptor varies as the sixth power of the distance between them (R) according to the following equation (Eq 1):

$$E={R_{0}}^{6}/{\left(R_{0}+R\right)}^{6}$$ (1)

R_0_ is the characteristic Förster distance for 50% energy transfer efficiency. The FRET pair (donor Cy3 and acceptor Cy5) used here has a calculated R_0_ of 53.5 Å. Three fluorescence measurements were performed using a Typhoon scanner equipped with the corresponding laser and filter settings: (1) donor emission (D) after donor excitation, (2) acceptor emission (A) after acceptor excitation and (3) FRET (F), which was observed by exciting the donor and measuring the emission from the acceptor.

When measuring FRET, extra intensity can be observed due to donor leakage and direct excitation of the acceptor. Two correction factors for donor leakage (χD) and direct acceptor excitation (χA) were determined and applied to the raw FRET scan. The χD and χA values were averaged from the quintuple reactions and are approximately 0.07 and 0.034, respectively. Furthermore, since protein binding events can change the fluorescence intensity, we determined the protein-dependent correction parameters σD and σA to correct the changes in fluorescence intensity between free DNA and protein-bound complexes. Using 12.5 μM protein, the σD value is 0.939 for the wild-type Sso7c4 and 0.932 for the C-terminally truncated mutant, and the σA value is 0.979 for the wild-type Sso7c4 and 0.955 for the C-terminally truncated mutant. Using these values, we calculated corrected FRET and fluorescence intensities (F_corr_ and D_corr,_ respectively) with Eqs 2 and 3, based on the corresponding raw signals (F and D). Finally, we used the corrected parameters to calculate the FRET efficiencies (Eq 4).

$$F_{corr}=\frac{F-(\chi D\times D)-(\chi A\times A)}{\sigma A}$$ (2)

$$D_{corr}=\frac{D}{\sigma D}$$ (3)

$$E=\left(F_{corr}\right)/\left(F_{corr}+D_{corr}\right)$$ (4)

## Supporting Information

S1 Fig Crystal structure of C-terminally truncated Sso7c4. (A) The crystal of C-terminally truncated Sso7c4 contains three homodimers in the asymmetric unit with the space group *P*3_1_. (B) In the trigonal crystal of C-terminally truncated Sso7c4, the α carbons of the three molecules in one asymmetric unit superimpose well. The r.m.s.d. values are 0.27 Å for 86 atoms and 0.34 Å for 79 atoms, represented by chain AB vs. chain CD and chain AB vs. chain EF, respectively. (C) The superposition of the α carbons of the wild-type and C-terminally truncated crystal structures and the NMR solution structure (2L66) is shown. By comparing the r.m.s.d. value of chain AB between the wild-type and C-terminally truncated structures, which is 0.35 Å for 85 atoms, the r.m.s.d. values between the coordinates of the wild-type and C-terminally truncated crystals and the coordinates of the NMR solution model are 1.56 Å for 92 atoms and 1.64 Å for 90 atoms, respectively. The crystal structures of the wild-type and C-terminally truncated proteins are similar to each other, but they have larger deviations from the NMR solution structure. The wild-type structure is shown in magenta, the C-terminally truncated structure is shown in blue, and the 2L66 structure is shown in green.
(TIF)

S2 Fig Intermolecular interactions of the Sso7c4 monomers at the dimerization interface. K45 in β4 and K45′ in β4′, together with D38 (D38′), S40 (S40′) and E41 (E41′), form a network of electrostatic interactions and water-mediated hydrogen bonds between the two monomers. The water molecules are drawn as cyan spheres. The hydrogen bonds are shown as yellow dashed lines.
(TIF)

S3 Fig Sulfate bound to the top surface of Sso7c4. The ionic interactions surrounding sulfate X203 are provided by the NH groups of N12′ and Q14′ in one monomer and Q14 in the other monomer. The hydroxyl group of S10′ forms a hydrogen bond with sulfate X203, which is only solvated by two water molecules. The water molecules are drawn as cyan spheres. The hydrogen bonds are shown as yellow dashed lines.
(TIF)

S4 Fig The fluorescence spectra of Sso7c4 and its DNA-binding curve. Fluorescence emission spectra of the intrinsic tryptophan residue (W53) of wild-type Sso7c4 (7.0 μM of homodimer) and its fluorescence quenching by incubation with the 20-bp dsDNA in 20 mM Tris-Cl, pH 7.5 at 25°C (left panel, selected spectra shown). The binding of Sso7c4 to the dsDNA was indicated by the quenching of the tryptophan (W53) fluorescence (345 nm) in the protein as a function of DNA concentration (right panel).
(TIF)

S5 Fig Raw EM images of nicked circular phiX174 plasmid with and without the Sso7c4. Overview raw images of phiX174 plasmid and Sso7c4-plasmid complexes visualized by EM. Nicked phiX174 plasmids were incubated with different stoichiometries (5 bp/dimer or 0.5 bp/dimer) of either the wild-type (wt) or C-terminally truncated (ctt) proteins. (A) Relaxed, circular phiX174 plasmid. (B) and (C) Wild-type Sso7c4-plasmid complex. (D) and (E) C-terminally truncated Sso7c4-plasmid complex. The scale bar represents 100 nm.
(TIF)

S6 Fig Images of a 384-well microplate after each of three scans. Solution-based FRET assays were performed in a 384-well borosilicate microplate using a Typhoon FluorImager to measure the effects of wild-type Sso7c4 and the C-terminally truncated mutant on the conformation of the 24-bp DNA. The fluorescence intensity of the donor (D) is shown in green, that of the acceptor (A) is shown in red and FRET (F) is shown in yellow.
(TIF)

S7 Fig Alignment of the Sso7c4 and histone H3 structures and structural comparison between histone H3 and HMfA. (A)The conserved R22 side chain of Sso7c4 is superimposed with R72 of histone H3 in the nucleosome (1KX5). The R11′ side chain of Sso7c4 is located in the minor groove, in which the R83 residue of histone H3 binds the DNA. (B) The superposition of the α carbons of the histone H3 and archaeal histone HMfA (1B67) crystal structures. The ribbon diagrams of the Sso7c4 dimer are colored in green and pink to represent each monomer. Histone H3 of the nucleosome is depicted as a blue ribbon. The archaeal histone HMfA is depicted as a cyan ribbon. The DNA duplex is shown in light orange. All arginine side chains are depicted as balls and sticks.
(TIF)

S1 Table Data collection and refinement statistics for the Sso7c4 crystals. (DOCX)

S2 Table Statistics of nucleoprotein complexes with different forms. (DOCX)
