# Supplementary figures and images for: The Arginine Pairs and C-Termini of the Sso7c4 from Sulfolobus solfataricus Participate in Binding and Bending DNA
Source: PLoS One. 2017 Jan 9;12(1):e0169627. doi: 10.1371/journal.pone.0169627 (PMC5222340; doi:10.1371/journal.pone.0169627)

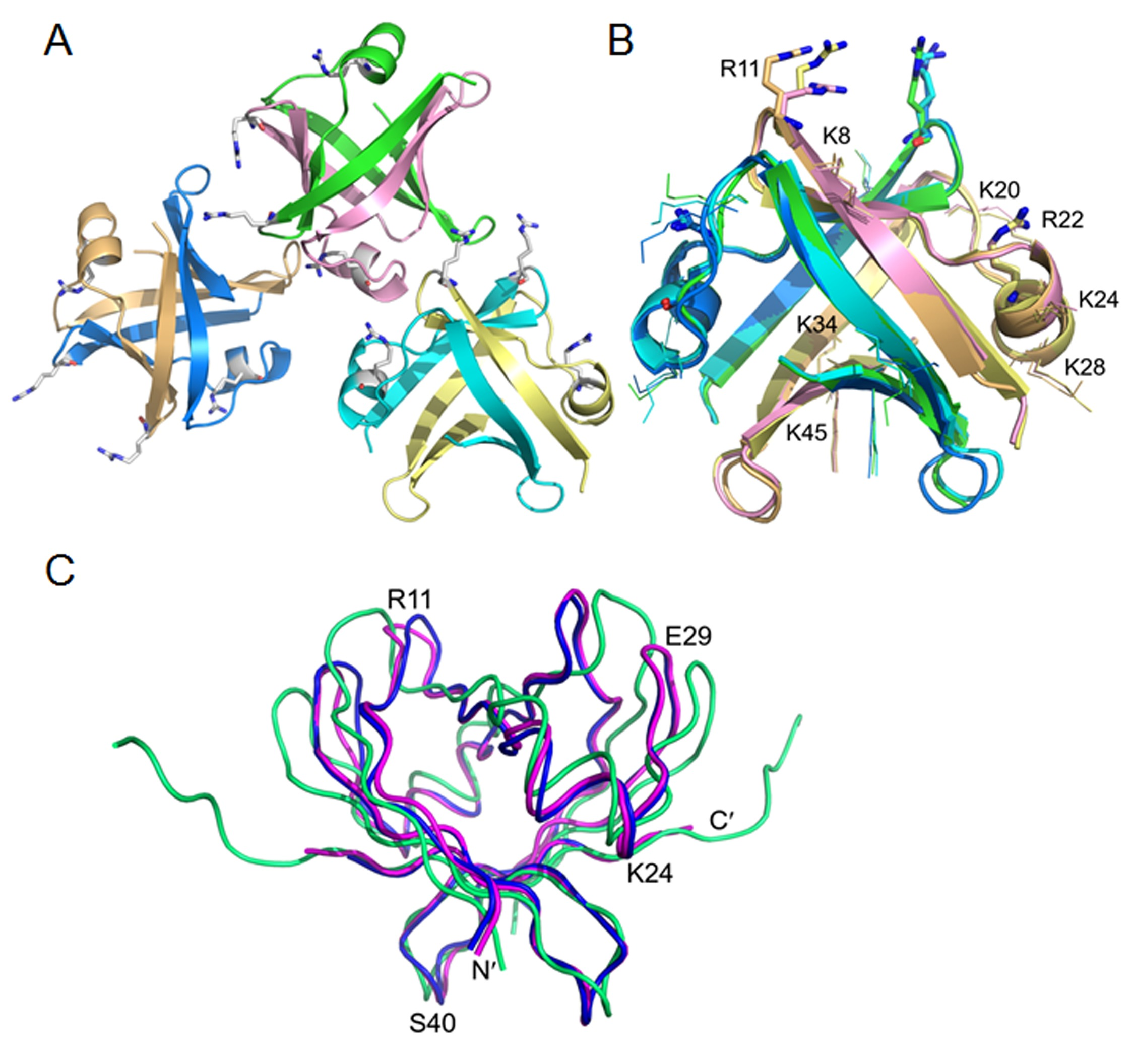

Supplement: S1 Fig — (A) The crystal of C-terminally truncated Sso7c4 contains three homodimers in the asymmetric unit with the space group P31. (B) In the trigonal crystal of C-terminally truncated Sso7c4, the α carbons of the three molecules in one asymmetric unit superimpose well. The r.m.s.d. values are 0.27 Å for 86 atoms and 0.34 Å for 79 atoms, represented by chain AB vs. chain CD and chain AB vs. chain EF, respectively. (C) The superposition of the α carbons of the wild-type and C-terminally truncated crystal structures and the NMR solution structure (2L66) is shown. By comparing the r.m.s.d. value of chain AB between the wild-type and C-terminally truncated structures, which is 0.35 Å for 85 atoms, the r.m.s.d. values between the coordinates of the wild-type and C-terminally truncated crystals and the coordinates of the NMR solution model are 1.56 Å for 92 atoms and 1.64 Å for 90 atoms, respectively. The crystal structures of the wild-type and C-terminally truncated proteins are similar to each other, but they have larger deviations from the NMR solution structure. The wild-type structure is shown in magenta, the C-terminally truncated structure is shown in blue, and the 2L66 structure is shown in green. (TIF) [file pone.0169627.s001.tif]

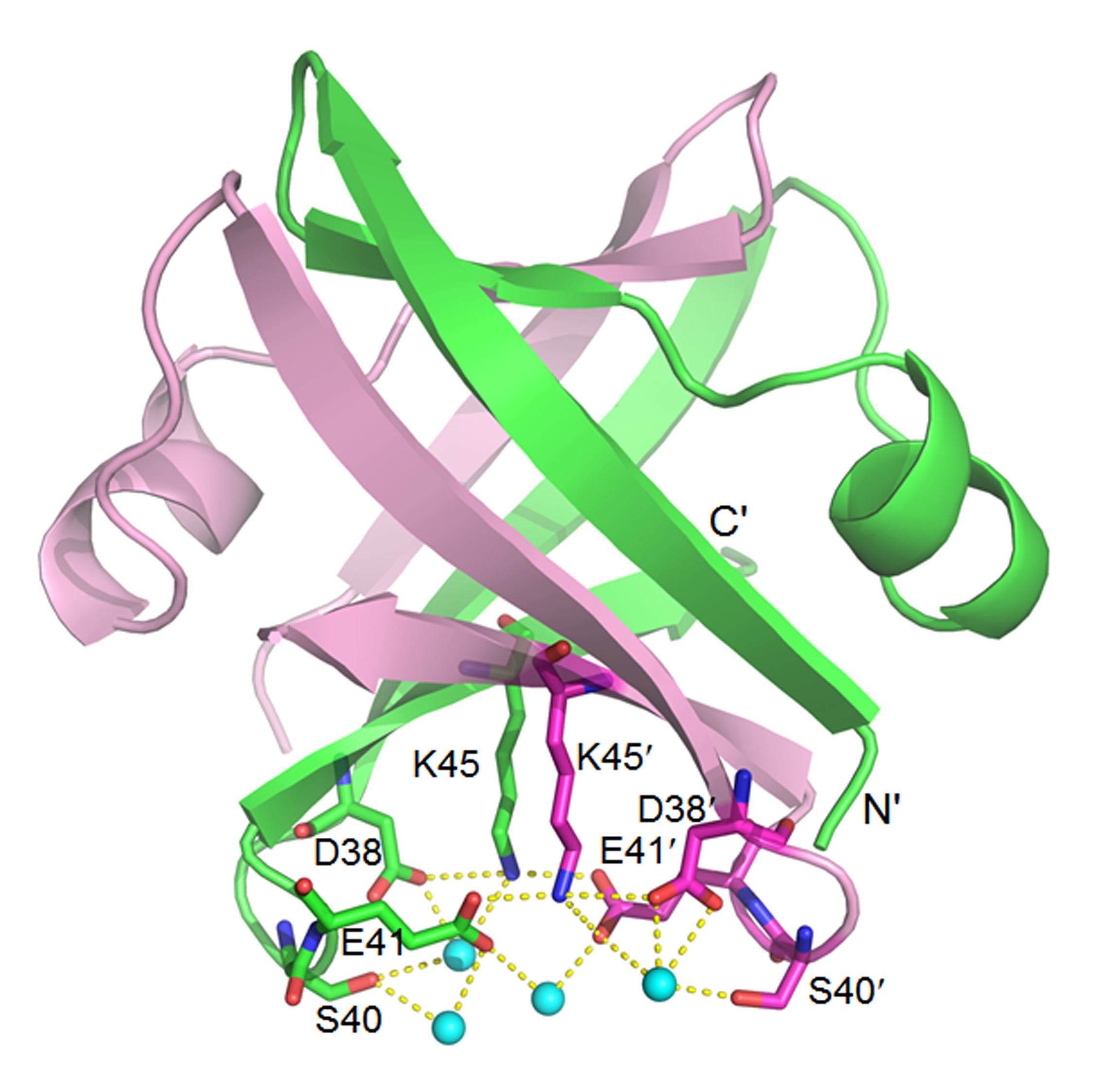

Supplement: S2 Fig — K45 in β4 and K45′ in β4′, together with D38 (D38′), S40 (S40′) and E41 (E41′), form a network of electrostatic interactions and water-mediated hydrogen bonds between the two monomers. The water molecules are drawn as cyan spheres. The hydrogen bonds are shown as yellow dashed lines. (TIF) [file pone.0169627.s002.tif]

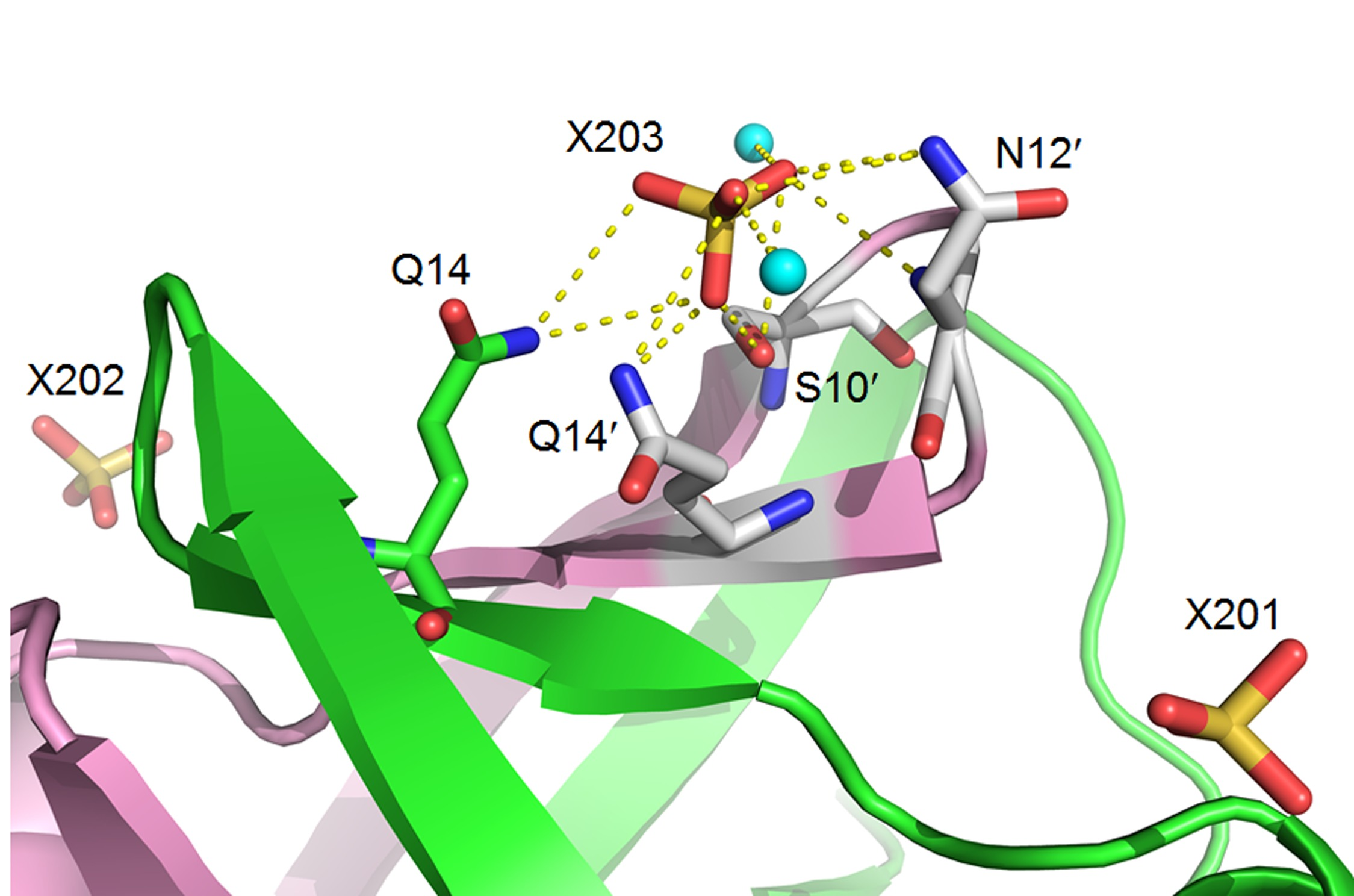

Supplement: S3 Fig — The ionic interactions surrounding sulfate X203 are provided by the NH groups of N12′ and Q14′ in one monomer and Q14 in the other monomer. The hydroxyl group of S10′ forms a hydrogen bond with sulfate X203, which is only solvated by two water molecules. The water molecules are drawn as cyan spheres. The hydrogen bonds are shown as yellow dashed lines. (TIF) [file pone.0169627.s003.tif]

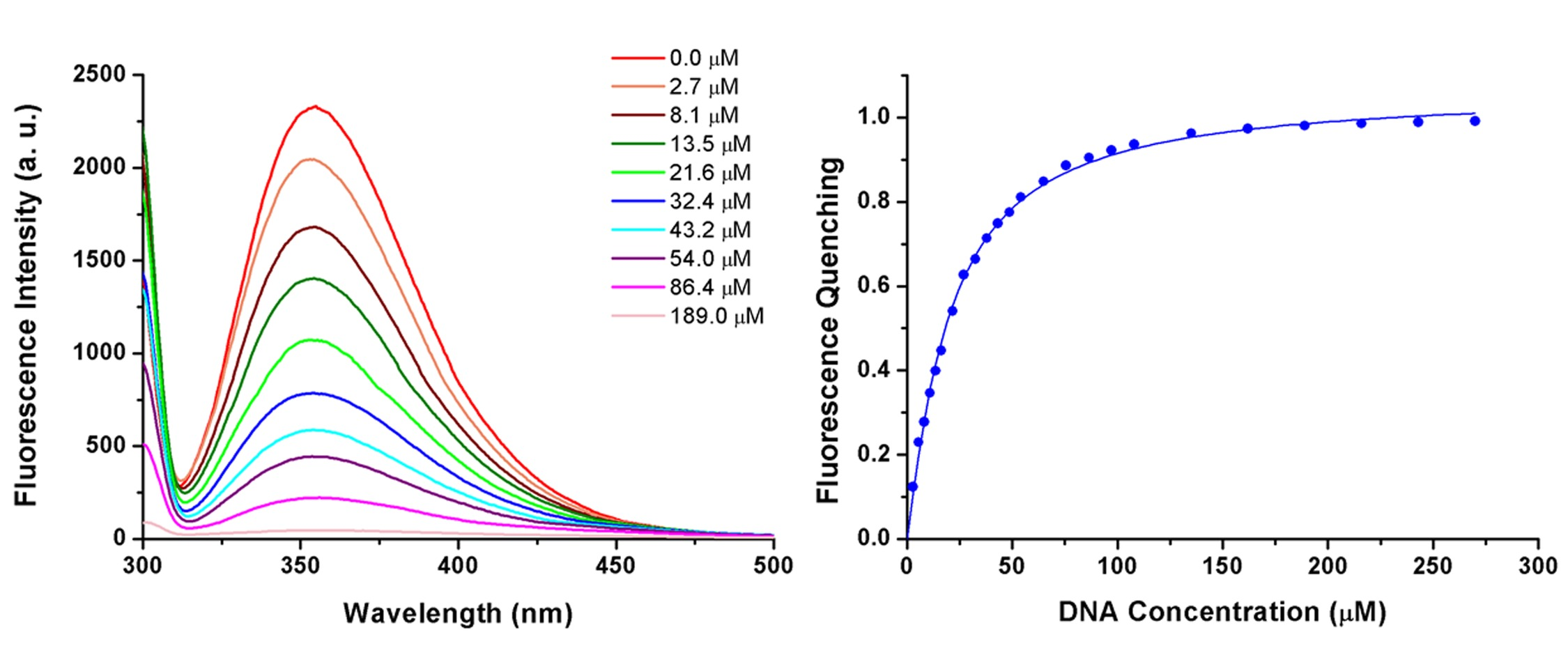

Supplement: S4 Fig — Fluorescence emission spectra of the intrinsic tryptophan residue (W53) of wild-type Sso7c4 (7.0 μM of homodimer) and its fluorescence quenching by incubation with the 20-bp dsDNA in 20 mM Tris-Cl, pH 7.5 at 25°C (left panel, selected spectra shown). The binding of Sso7c4 to the dsDNA was indicated by the quenching of the tryptophan (W53) fluorescence (345 nm) in the protein as a function of DNA concentration (right panel). (TIF) [file pone.0169627.s004.tif]

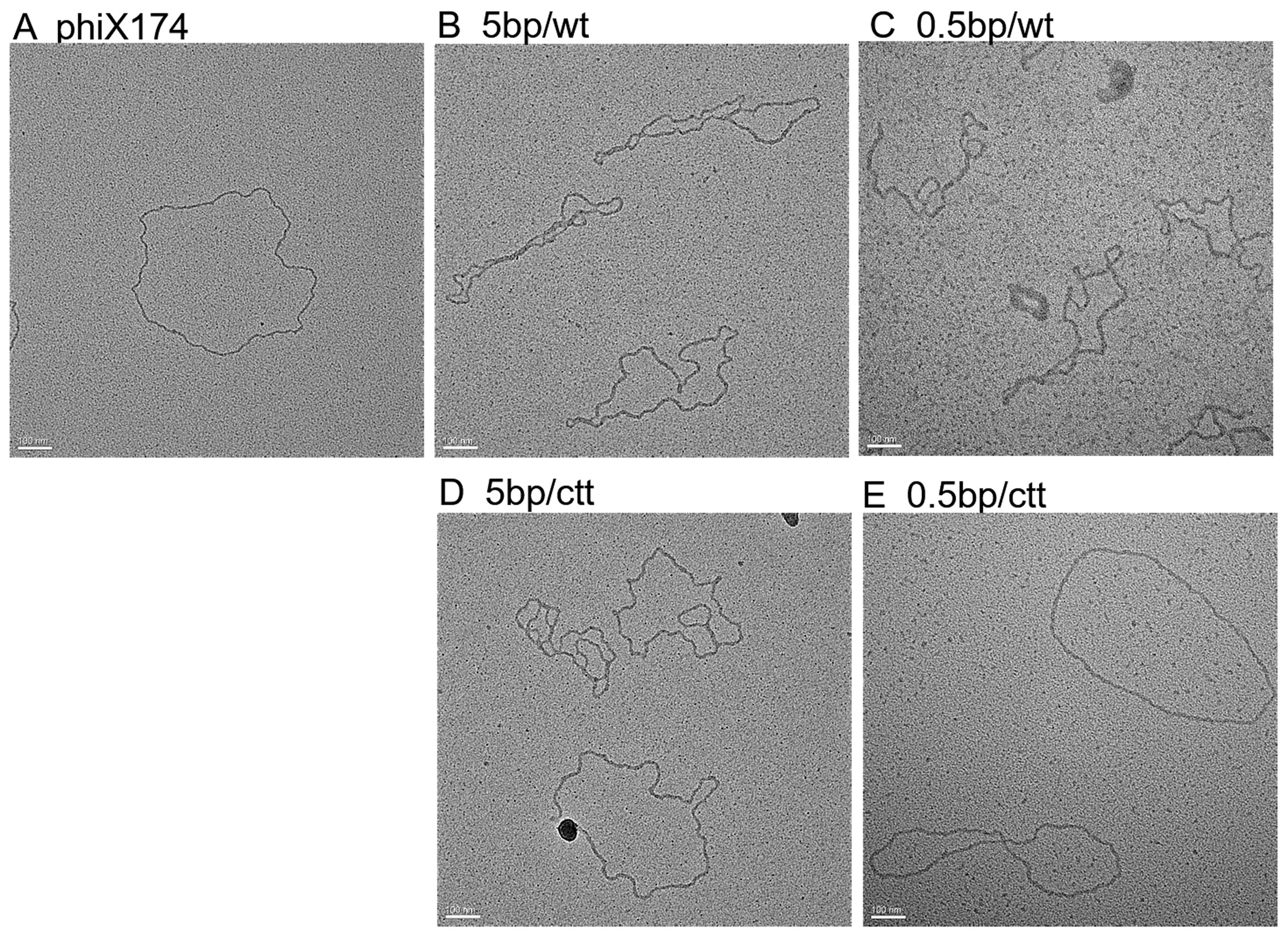

Supplement: S5 Fig — Overview raw images of phiX174 plasmid and Sso7c4-plasmid complexes visualized by EM. Nicked phiX174 plasmids were incubated with different stoichiometries (5 bp/dimer or 0.5 bp/dimer) of either the wild-type (wt) or C-terminally truncated (ctt) proteins. (A) Relaxed, circular phiX174 plasmid. (B) and (C) Wild-type Sso7c4-plasmid complex. (D) and (E) C-terminally truncated Sso7c4-plasmid complex. The scale bar represents 100 nm. (TIF) [file pone.0169627.s005.tif]

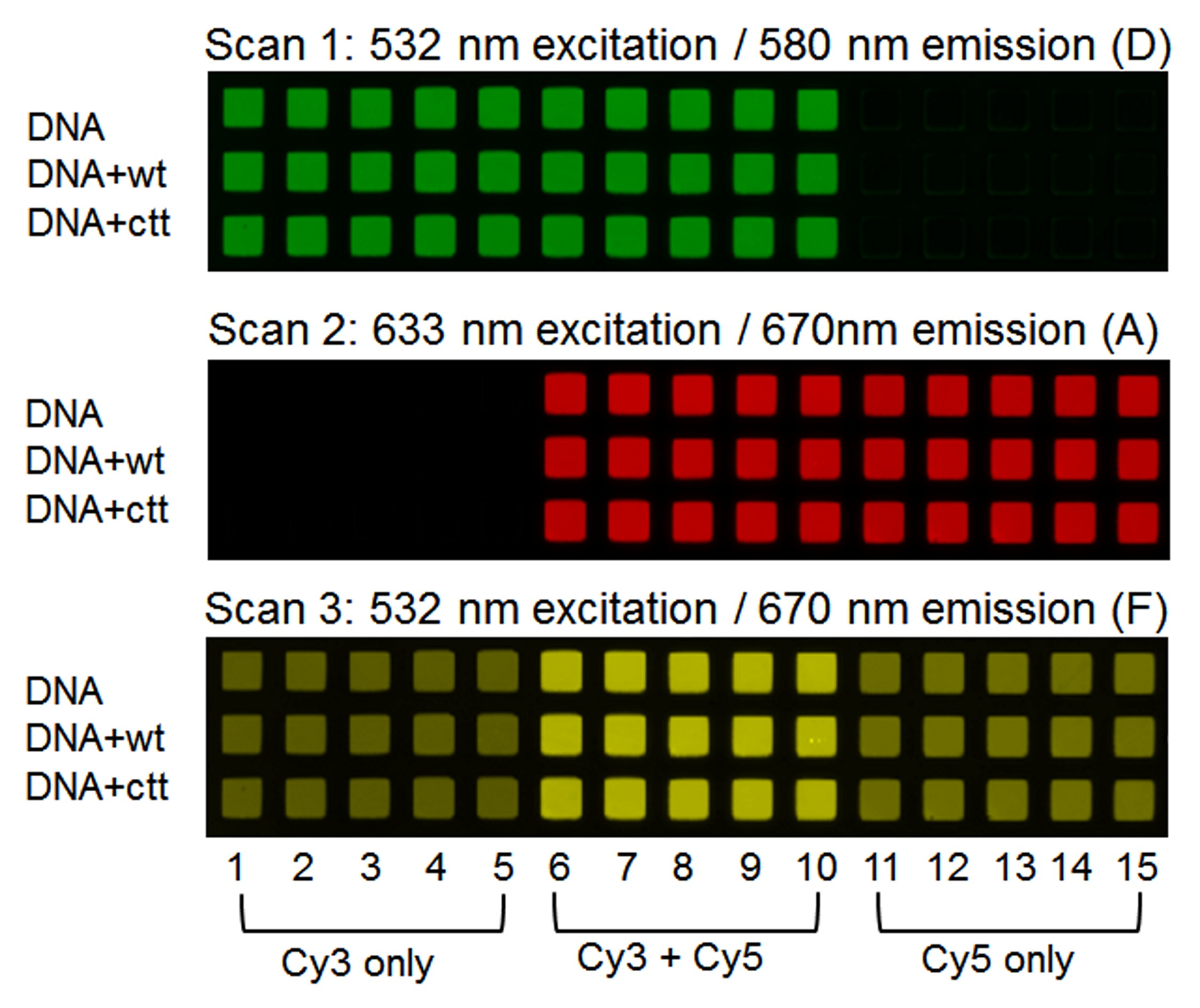

Supplement: S6 Fig — Solution-based FRET assays were performed in a 384-well borosilicate microplate using a Typhoon FluorImager to measure the effects of wild-type Sso7c4 and the C-terminally truncated mutant on the conformation of the 24-bp DNA. The fluorescence intensity of the donor (D) is shown in green, that of the acceptor (A) is shown in red and FRET (F) is shown in yellow. (TIF) [file pone.0169627.s006.tif]

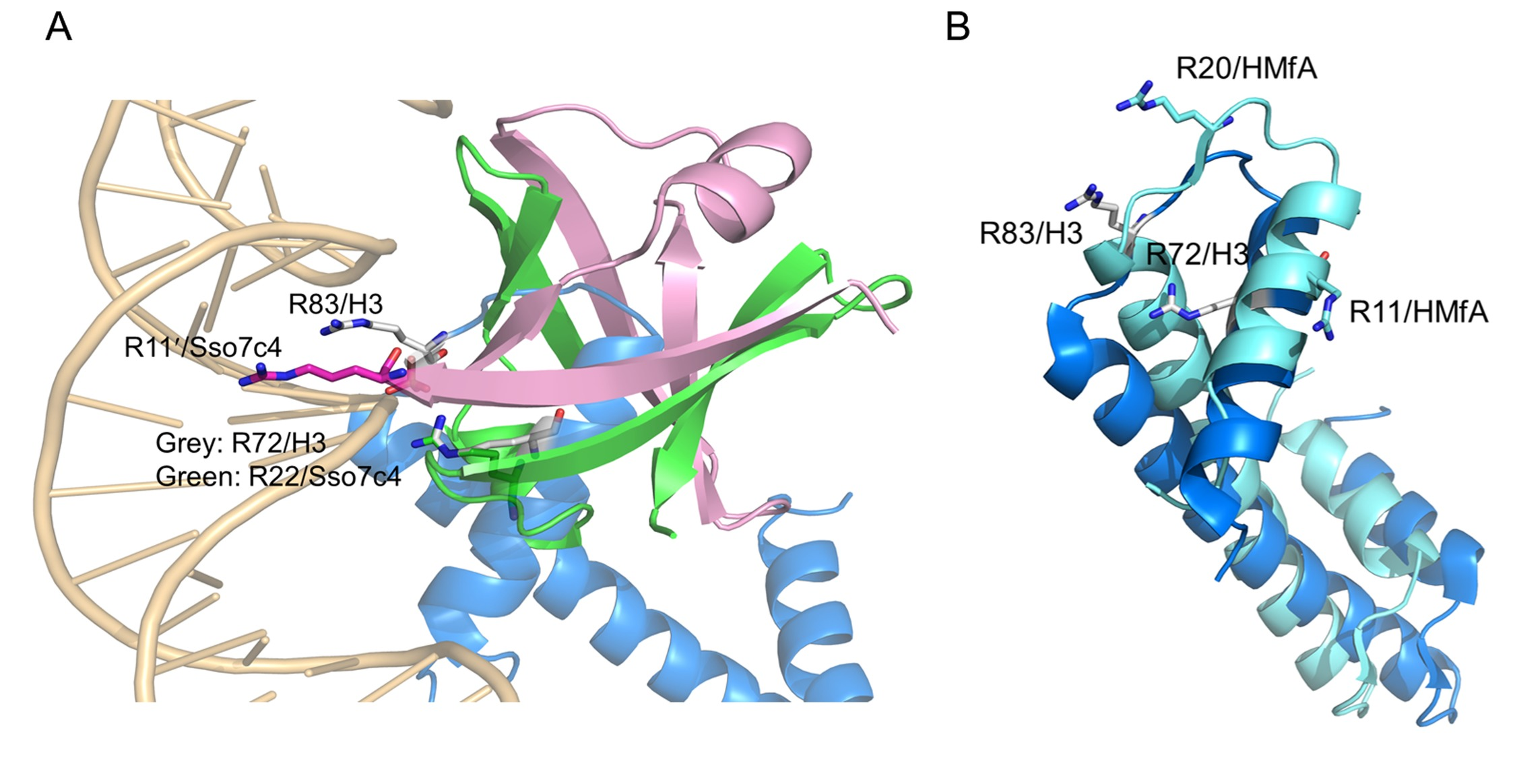

Supplement: S7 Fig — (A)The conserved R22 side chain of Sso7c4 is superimposed with R72 of histone H3 in the nucleosome (1KX5). The R11′ side chain of Sso7c4 is located in the minor groove, in which the R83 residue of histone H3 binds the DNA. (B) The superposition of the α carbons of the histone H3 and archaeal histone HMfA (1B67) crystal structures. The ribbon diagrams of the Sso7c4 dimer are colored in green and pink to represent each monomer. Histone H3 of the nucleosome is depicted as a blue ribbon. The archaeal histone HMfA is depicted as a cyan ribbon. The DNA duplex is shown in light orange. All arginine side chains are depicted as balls and sticks. (TIF) [file pone.0169627.s007.tif]
