# Supplementary material for: The Arginine Pairs and C-Termini of the Sso7c4 from Sulfolobus solfataricus Participate in Binding and Bending DNA
Source: PLoS One. 2017 Jan 9;12(1):e0169627. doi: 10.1371/journal.pone.0169627 (PMC5222340; doi:10.1371/journal.pone.0169627)
Supplement: S1 Table — (DOCX) [file pone.0169627.s008.docx]

**S1 Table**. Data collection and refinement statistics for the Sso7c4 crystals.

|  | **Sso7c V21M**  **Se high remote** | **Sso7c V21M**  **Se inflection** | **wt Sso7c4** | **ctt Sso7c4** |
| --- | --- | --- | --- | --- |
| *Data collection* |  |  |  |  |
| Light source | NSRRC BL13B1 | NSRRC BL13B1 | FR-E+ Super Bright/R-AXIS HTC | NSRRC BL13C1 |
| Wavelength (Å) | 0.9635 | 0.9789 | 1.54 | 0.9760 |
| Resolution (Å) | 30.0 – 2.01  (2.08-2.01) | 30.0 – 2.05  (2.12-2.05) | 30.0 - 1.63  (1.69 - 1.63) | 30.0 – 1.40  (1.45 - 1.40) |
| Space group | *P*2_1_2_1_2_1_ | *P*2_1_2_1_2_1_ | *P*2_1_2_1_2_1_ | *P*3_1_ |
| Unit-cell *a,* *b,* *c* (Å)  α, β, γ (°) | 34.54, 45.25, 59.68  90.00, 90.00, 90.00 | 34.54, 45.25, 59.68  90.00, 90.00, 90.00 | 41.46, 48.54, 55.99  90.00, 90.00, 90.00 | 49.33, 49.33, 82.47  90.00, 90.00, 120.00 |
| No. of unique reflections | 6529 (614) | 6191 (600) | 14365 (1344) | 43748 (4408) |
| Redundancy | 5.5 (4.7) | 5.5 (5.0) | 8.0 (8.6) | 4.5 (4.8) |
| Completeness (%) | 97.9 (94.3) | 98.2 (98.8) | 97.7 (94.1) | 99.2 (100.0) |
| Mean *I*/*σ(I)* | 32.4 (12.9) | 31.49 (14.29) | 44.7 (11.3) | 30.6 (3.0) |
| *R*_merge_ (%)^a^ | 4.3 (12.1) | 5.1 (11.0) | 4.6 (29.1) | 5.5 (48.5) |
| *Refinement* |  |  |  |  |
| No. of chain(s) |  |  | 2 | 6 |
| No. of reflections |  |  | 13605 (1008) | 41484 (3223) |
| R_work_ (95% of data) |  |  | 0.178 (0.234) | 0.168 (0.180) |
| R_free_ (5% of data) |  |  | 0.229 (0.287) | 0.197 (0.246) |
| r.m.s.d. bonds (Å) |  |  | 0.027 | 0.023 |
| r.m.s.d. angles (°) |  |  | 2.6 | 2.3 |
| Mean B (Å^2^) / atoms |  |  |  |  |
| Protein atoms |  |  | 19.2 / 772 | 17.1 / 2255 |
| Water molecules |  |  | 36.3 / 146 | 26.8 / 311 |
| PEG atoms |  |  | 48.2 / 24 |  |
| Ion atoms |  |  | 36.9 / 15 |  |
| *Ramachandran plot (%)* |  |  |  |  |
| Most favored (%) |  |  | 100.0 | 99.6 |
| Allowed (%) |  |  | 0.0 | 0.4 |
| Disallowed (%) |  |  | 0.0 | 0.0 |
| *PDB ID code* |  |  | *5ITJ* | *5ITM* |

The values in parentheses indicate the highest resolution shell.

^a^ *R*_merge_ = ∑*_hkl_*∑_i_|*I*_i_(*hkl*)-<*I*(*hkl*)>| / ∑*_hkl_*∑_i_*I*_i_(*hkl*).
