# Supplementary material for: The Arginine Pairs and C-Termini of the Sso7c4 from Sulfolobus solfataricus Participate in Binding and Bending DNA
Source: PLoS One. 2017 Jan 9;12(1):e0169627. doi: 10.1371/journal.pone.0169627 (PMC5222340; doi:10.1371/journal.pone.0169627)
Supplement: S2 Table — (DOCX) [file pone.0169627.s009.docx]

**Table S2.** Statistics of nucleoprotein complexes in different forms.

| EM sample | Bridged complex | Non-bridged complex |
| --- | --- | --- |
| wild-type Sso7c4-plasmid  (5 bp/dimer, n=134) | 93% | 7% |
| wild-type Sso7c4-plasmid  (0.5 bp/dimer, n=86) | 98%  (complete bridged, 15%) | 2% |
| C-terminally truncated Sso7c4-plasmid  (5 bp/dimer, n=178) | 70% | 30% |
| C-terminally truncated Sso7c4-plasmid  (0.5 bp/dimer, n=109) | 33% | 67%  (more open form) |

n: Number of molecules
